# Systems Level Analysis of the Yeast Osmo-Stat

**DOI:** 10.1038/srep30950

**Published:** 2016-08-12

**Authors:** Soheil Rastgou Talemi, Carl-Fredrik Tiger, Mikael Andersson, Roja Babazadeh, Niek Welkenhuysen, Edda Klipp, Stefan Hohmann, Jörg Schaber

**Affiliations:** 1 Institut für Experimentelle Innere Medizin, Medizinische Fakultät, Otto-von-Guericke-Universität, Magdeburg, Germany; 2 Division of Theoretical Systems Biology, German Cancer Research Center (DKFZ), Heidelberg, Germany; 3 Department of Chemistry and Molecular Biology, University of Gothenburg, SE-405 30 Göteborg, Sweden; 4 Theoretical Biophysics, Humboldt Universität zu Berlin, Berlin, Germany; 5 Department of Biology and Biological Engineering, Chalmers University of Technology, SE-412 96 Göteborg, Sweden

## Abstract

Adaptation is an important property of living organisms enabling them to cope with environmental stress and maintaining homeostasis. Adaptation is mediated by signaling pathways responding to different stimuli. Those signaling pathways might communicate in order to orchestrate the cellular response to multiple simultaneous stimuli, a phenomenon called crosstalk. Here, we investigate possible mechanisms of crosstalk between the High Osmolarity Glycerol (HOG) and the Cell Wall Integrity (CWI) pathways in yeast, which mediate adaptation to hyper- and hypo-osmotic challenges, respectively. We combine ensemble modeling with experimental investigations to test in quantitative terms different hypotheses about the crosstalk of the HOG and the CWI pathways. Our analyses indicate that for the conditions studied i) the CWI pathway activation employs an adaptive mechanism with a variable volume-dependent threshold, in contrast to the HOG pathway, whose activation relies on a fixed volume-dependent threshold, ii) there is no or little direct crosstalk between the HOG and CWI pathways, and iii) its mainly the HOG alone mediating adaptation of cellular osmotic pressure for both hyper- as well as hypo-osmotic stress. Thus, by iteratively combining mathematical modeling with experimentation we achieved a better understanding of regulatory mechanisms of yeast osmo-homeostasis and formulated new hypotheses about osmo-sensing.

A change in ambient osmolarity is a commonly encountered environmental challenge for microorganisms. Yeast lives on fruits or other plant materials whose sap can cause severe hyper-osmotic conditions whereas rain may lead to sudden hypo-osmotic conditions. Changes in the environmental osmotic conditions result passively in cell volume changes by water flows, which rapidly equilibrate external and internal water potential differences^1^,^2^,^3^. In order to restore the optimal cell volume and the internal water balance, yeasts have to adapt their internal osmolarity to the new external conditions. In the yeast *Saccharomyces cerevisiae*, two mitogen-activated protein kinase (MAPK) pathways, the High Osmolarity Glycerol (HOG) pathway^4^ and Cell Wall Integrity (CWI) pathway^5^, have been implicated in the adaptation to cell volume changes.

The HOG pathway mediates cell volume and turgidity restoration and adaptation to high osmolarity condition by engaging in several activities; cell cycle arrest, changes enzymes activities, glycerol channel closure and gene expression^4^ as well as complex metabolic adaptation processes^6^ leading to both production and retention of the osmolyte glycerol. The best-studied regulator of this process is the MAPK Hog1, which acts as the effector kinase in a highly conserved three-tiered MAPK cascade. Upon cell shrinkage, Hog1 is phosphorylated and activates transcription, which in turn up-regulates the production of enzymes producing glycerol as an intracellular osmolyte. In addition, it is believed that the phosphorylated Hog1 closes the glycerol channel Fps1 by phosphorylating and displacing its positive regulators Rgc1/2^7^,^8^. The increase in internal osmolarity forces water back into the cell mediating restoration of pre-stress volume. In addition to experimental investigations on the HOG pathway, mathematical modelling and simulations have added substantially to our understanding about the information processing, the role of multiple feedbacks and the importance of two parallel signaling branches in this signaling system^3^,^9^,^10^,^11^,^12^,^13^.

The CWI pathway is activated in response to different stimuli including heat stress, pheromone-induced morphogenesis and hypo-osmotic shock (HYPOS)^5^. Proteins in the plasma membrane detect cell wall stress and activate several proteins, which eventually leads to the activation of the MAPK Slt2. Just like Hog1 in the HOG pathway, Slt2 is the effector kinase of the CWI pathway. It initiates both transcriptional and post- transcriptional responses to remodel the cell wall following the above-mentioned stimuli^5^.

Although MAPK signaling pathways are conceived as being rather signal-specific, there is growing evidence that these pathways constitute a strongly connected network and that pathways communicate with each other^14^,^15^,^16^,^17^,^18^. Baltanás *et al*. suggested that Slt2 and Hog1 cooperate at the level of the Fps1 aquaglyceroporin, the main channel for glycerol efflux from the cell, in response to pheromone induction^15^. Although regulation of Fps1 through its regulators, Rgc1/2, has been identified to be Hog1-dependent^7^,^8^, Slt2 mediated Fps1 regulation is poorly understood. Bermejo and Garcia suggested an inhibitory effect of Hog1 and Slt2 on each other’s activation^19^,^20^.

In order to address the mechanism of a hypothetical crosstalk between the HOG and the CWI pathways, we simultaneously investigated the response of these pathways to both hyper- and hypo-osmotic challenges. To this end, we combined an ensemble modelling approach with experimental investigation of the HOG and CWI response to changes in environmental osmolarity. We implemented a number of different mathematical models representing different hypotheses about the interactions of the two pathways and selected the best supported model using an information-theoretic approach. According to the information theory paradigm, the selected model is believed to best approximate the true system in comparison to other models in the ensemble^21^. The best approximating model was used to design experiments that were able to gain further evidence for the supported hypotheses. These dedicated follow-up experiments corroborated our model. Our combined modelling and experimental investigation indicated that:

- In contrast to the HOG pathway, whose activation relies on a fixed volume threshold, the CWI pathway employs an adaptive mechanism with a variable volume-dependent threshold.
- There is no or little direct crosstalk between the HOG and CWI pathways for the conditions explored here.
- The HOG pathway mediates adaptation of cellular osmotic pressure for both hyper- as well as hypo-osmotic stress, whereas the CWI pathway does not seem to directly affect internal osmolarity.

Taken together, our approach revealed new insight into the yeast osmoadaptive mechanisms and the communication mechanisms between the HOG and CWI pathways.

## Results

### Systems analysis strategy

**Figure 1:**
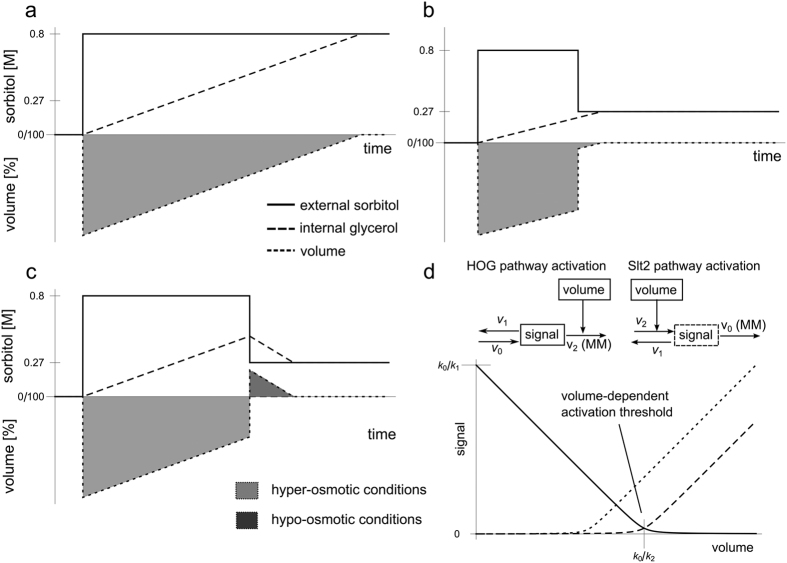
Hyper-hypo-shock concept and generic signal functions. (**a**–**c**) Conceptual illustration of the experimental setup for external osmolarity (solid line), and corresponding theoretical internal glycerol (dashed line) and volume (dotted line). (**a**) Single hyper-shock of 0.8 M sorbitol induces a sudden volume decrease with subsequent volume recovery as internal glycerol increases. (**b**) Single hyper-osmotic shock of 0.8 M sorbitol induces a sudden volume decrease with subsequent volume recovery as internal glycerol increases. Subsequent decrease to 0.27 M external sorbitol induces a sudden volume increase. Shift to 0.27 M sorbitol does not induce a hypo-osmotic shock, as the sudden volume increase does not exceed initial volume. (**c**) Single hyper-osmotic shock of 0.8 M sorbitol induces a sudden volume decrease with subsequent volume recovery as internal glycerol increases. Subsequent decrease to 0.27 M external sorbitol induces a sudden volume increase. Shift to 0.27 M sorbitol induces a hypo-shock, because the sudden volume increase exceeds initial volume. (**d**) Wiring schemes of the signaling module of the HOG and the CWI pathways with corresponding steady states as a function of volume. The parameters *k*_0_ and *k*_2_, defining the volume threshold upon which pathway activation is triggered, are the rate constants for reactions *v*_0_ and *v*_2_, respectively. Solid and dashed lines indicate Hog1 and Slt2 activation respectively. The dotted line indicates dynamic threshold regulation via a hypothetical sensitizing entity. MM: Michaelis-Menten kinetics. Mathematical details are provided in the Supplementary Material.

To study possible crosstalk and cooperation mechanisms between the HOG and the CWI pathways and CWI pathway regulation, upon osmotic stress, we subjected *S. cerevisiae* to various hyper and hyper-hypo-osmotic shocks regimes. A conceptual overview of external and internal changes in osmolarity as well as volume changes is depicted in Fig. 1. A sudden increase in external osmolarity (solid lines in Fig. 1a–c) leads to a corresponding drop in cell volume (dotted lines in Fig. 1a–c). The cell restores its volume by actively increasing internal osmolarity using glycerol as an osmolyte (dashed lines in Fig. 1a–c), this forces water back into the cell leading to volume restoration (dotted lines in Fig. 1a–c). For simplicity, this point is indicated in Fig. 1a–c, when internal glycerol is equal to the external osmolarity. However, at osmotic homeostasis there is always an osmotic gradient between inside and outside of the cell, which is balanced by the turgor pressure^1^,^2^.

In the standard experiment, we always started with a hyper-osmotic shock of 0.8M sorbitol at time *t* = 0 min and continued with dilutions, where we varied both the time (45 s, 90 s, 4 min, 14 min, 30 min, 45 min) and the final external sorbitol concentration (0.27, 0.4, 0.5 M external sorbitol) (see Materials and Methods, Hypo osmotic shocks induction). Subsequently, we simultaneously monitored the phosphorylation state of Hog1 and Slt2. Following dilution, intracellular glycerol concentration decreases via Fps1 mediated diffusion^22^. The effect of a dilution depends on the final external osmolarity as well as on the internal osmolarity at the time of dilution represented by the cell volume. When the dilution to lower sorbitol levels comes soon after the hyper-osmotic shock, the new cell volume might still be below the initial volume, because internal osmolarity did not yet increase to sufficient levels (Fig. 1b). In this situation, lower sorbitol levels would only relieve the hyper-osmotic shock conditions and reduce adaptation time (light gray areas in Fig. 1a–c). Only when dilution to lower external sorbitol is done when internal glycerol is sufficiently high, such that the decrease in external osmolarity and concomitant volume increase would render the newly acquired volume above the initial volume (Fig. 1c, dark gray area), a hypo-osmotic shock is prompted. Under these conditions, the cell has to decrease internal glycerol levels to restore initial pre-stress volume (Fig. 1c).

**Figure 2:**
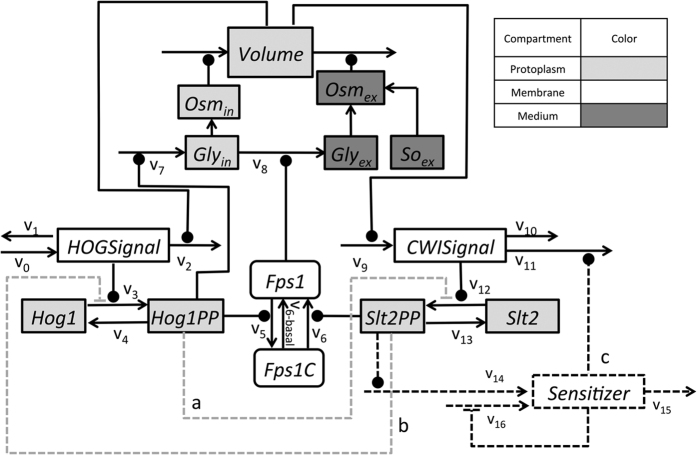
Alternative model structures. Dashed lines indicate alternative components defining different model candidates. The best ranked model included the dark dashed lines. Three different sources of variation were implemented, each of them could adopt two different setups, and consequently 8 different model combinations were achievable: (**a**) Activated Hog1 inhibits the Slt2 activation/phosphorylation (reaction *v*_12_), (**b**) Activated Slt2 inhibits the Hog1 activation/phosphorylation through (reaction *v*_3_) and (**c**) a Slt2PP-dependent *Sensitizer* decreases *CWISignal (v*_11_). We primarily started with four models and included the 3^rd^ source of variation “c” later on, as explained in the text. *Gly, Osm*, and *So* represent Glycerol, Osmotic pressure, and Sorbitol, respectively.

To rationalize the experimental results and to analyze the interactions between the HOG and CWI pathways we implemented four simple mathematical models representing different hypothesis about possible crosstalk mechanisms, which were formulated in the literature. These mathematical models represent combinations of ordinary differential equations (ODEs) and algebraic equations (see Materials and Methods, Ensemble modelling of the hyper-hypo-osmotic shock response). All four models employed a fixed activation threshold for both the HOG pathway as well as the CWI pathway (Fig. 1d; solid and dashed lines, respectively, Fig. 2; models without *v*_14_, *v*_15_ and *v*_16_). A fixed threshold translates into basal activation at the initial volume and quasi-linear activation upon volume decrease below or increase above the initial volume, i.e., the activation threshold for the HOG and the CWI pathway, respectively. Finally, after calibration of models to experimental data, we conducted model selection using Akaike Information Criterion corrected for small sample size (*AICc*) (see Materials and Methods, Model Inference and Selection Strategy). Model ranking resulted in selection of the simplest model, with an *AICc*-weight of 0.92 (Table S1). We repeated the model ranking by fitting all experimental data leading to the same result (Table S2). Simulation of the best ranked model showed good agreement with experimental data used for both parameter estimation and prediction (Figures S1 and S2).

### HOG pathway activation/deactivation dynamics are well captured for all models

The Hog1 phosphorylation dynamics could well be described and predicted for all tested models (Figures S1 and S2). This was expected, because very similar models of the HOG pathway have been shown to be able to describe hyper-osmotic shock responses. Remarkably, also the measured hypo-osmotic shock dynamics of activated Hog1 could be captured well. Therefore we focused on the less well studied Slt2 response.

### Model with fixed Slt2 activation threshold cannot explain the data

Neither the best ranked model nor the other models in the ensemble were able to reproduce Slt2 activation when the hypo-osmotic shock was applied 4 min after the hyper-osmotic shock (Figure S1b, Table S1).

Slt2 activation upon external osmolarity reduction crucially depends on the rate of glycerol accumulation (Fig. 1a–c). Therefore, we speculated that the lack of ability to reproduce the Slt2 activation, upon hypo-osmotic shock 4 min after the hyper-osmotic shock, is due to insufficient glycerol accumulation in the model. To test this idea, we simulated the same experimental condition using the model with twice the glycerol production rate. This time, the model simulations reproduced the Slt2 activation (Figure S3b). However, with such an increase in glycerol production rate, simulated volume and predicted internal glycerol for the 0.8 M hyper-osmotic shock scenario could no longer be reproduced (Figure S3c,d). Thus, the observed Slt2 activation does not correspond to the measured glycerol accumulation rate and volume dynamics. Consequently, the mechanism of Slt2 activation must be different from the ones assumed, at least at this particular time point.

### Slt2 activation threshold is dynamically regulated by a sensitizing mechanism

Although we tested different combinations of hypothetical interactions between Slt2 and Hog1, none of those combinations were able to explain 4 min Slt2 activation data (Figure S3b, Tables 1 and S1). We therefore hypothesized that the volume activation threshold for Slt2 is not fixed, but is itself adaptive in a way that it decreases with decreasing volume (Fig. 1d, right wiring scheme, dotted line). There are various ways to achieve such a behavior in models. Taking the structure of CWI pathway activation as depicted in Fig. 1d, the activation threshold is given by the rate constants of reaction *v*_0_ and *v*_2_, i.e. *k*_0_/*k*_2_. Thus, to decrease the activation threshold one can either decrease *k*_0_ or increase *k*_2_. Here, for the lack of knowledge about the true mechanisms activating the CWI pathway, we arbitrarily adopted the former mechanisms. We assumed that the Slt2 activation threshold depends on the state of a hypothetical sensitizer that in turn depends on Slt2 activity and affects *k*_0_ (Fig. 1d, right wiring scheme, dotted line) or *k*_11_ in the complete models (Fig. 2).

**Figure 3:**
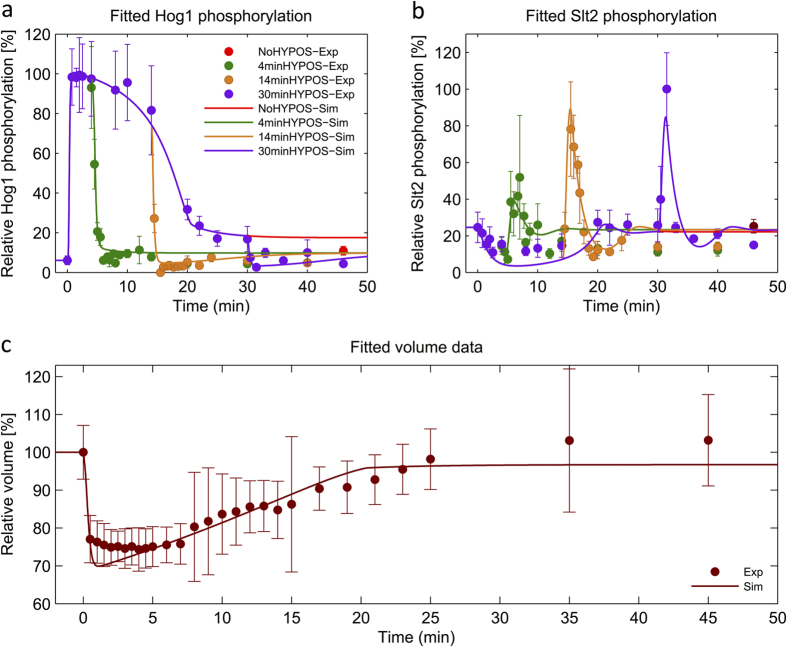
Reproduction of experimental data dedicated for parameter estimation using model with sensitized negative feedback. Relative Hog1 and Slt2 phosphorylation data and relative single cell volume measurements, used for models parameters estimation, are plotted versus time. Simulations were done using the best ranked model from the ensemble of models with sensitized negative feedback. Solid lines show model simulations and filled circles (•) show the experimental data (Mean ± SD (n = 3)). (**a**) Comparison between Hog1 phosphorylation data and respective simulation for 0.8 M sorbitol shock only (NoHYPOS-Exp) and 4′, 14′ and 30′ hypo-shock experiments using the best ranked model (4 minHYPOS, 14 minHYPOS, 30 minHYPOS, respectively). (**b**) Comparison between Slt2 phosphorylation data and its simulation for 0.8 M sorbitol shock only, 4′, 14′, 30′ hypo-shock using best ranked model. The selected model can reproduce the 4′ Slt2 activation. (**c**) Comparison between the relative value of single cell volume measurement and its simulation upon 0.8 M sorbitol shock. The same color code was used for panel a,b.

**Figure 4:**
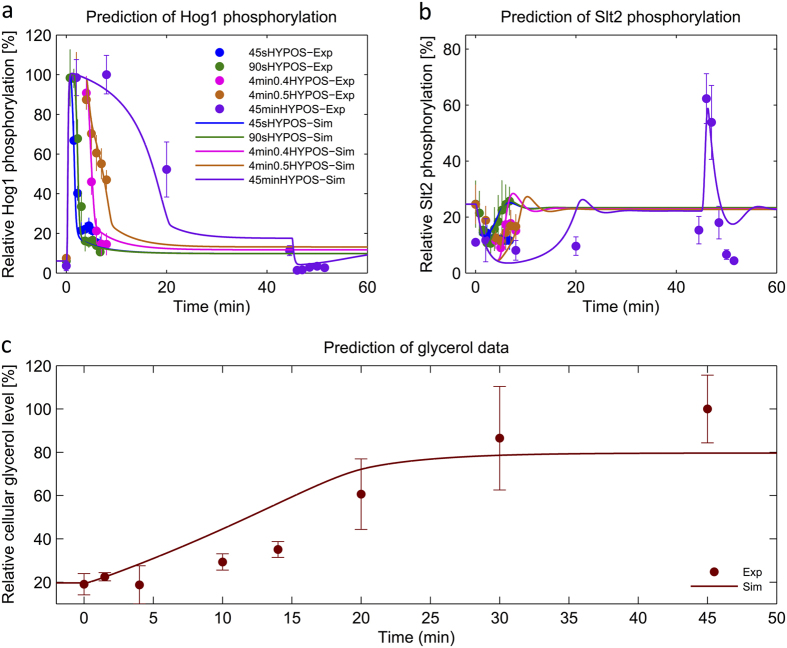
Reproduction of experimental data dedicated for prediction using model with sensitized negative feedback. Relative Hog1 and Slt2 phosphorylation data and relative value of cellular glycerol measurements, used for prediction, are plotted versus time. Simulations were done using the best ranked model from the ensemble of models with sensitized negative feedback. Solid lines show model simulations and filled circles (•) show the experimental data (Mean ± SD (n = 3)). (**a**) Comparison between Hog1 phosphorylation data and its simulation for 0.8 M sorbitol shock with subsequent dilution to 0.27 M sorbitol at 45″, 90″ and 45′ (45SecHYPO-Exp, 90SecHYPO-Exp, 45 minHYPO-Exp) and 0.8 M sorbitol shock with subsequent dilution to 0.5 and 0.4 M sorbitol at 4′ (4 min0.5HYPO-Exp, 4 min0.4HYPO-Ex) using the best ranked model. (**b**) Comparison between Slt2 phosphorylation data and its simulation for 0.8 M sorbitol shock with subsequent dilution to 0.27 M sorbitol at 45″, 90″ and 45′ and 0.8 M sorbitol shock with subsequent dilution to 0.5 and 0.4 M sorbitol at 4′ using the best ranked model. (**c**) Comparison between the relative value of intracellular glycerol content for 0.8 M sorbitol shock and its simulation. We used same color code for panel a,b.

With this modification, we tested all model alternatives again (with and without sensitizer in Fig. 2) and we selected the best ranked model (Table 1). We repeated the model ranking by including all experimental data for parameter estimation which led to the same result (Table S3). We also conducted identifiability analysis for this new selected model. This analysis indicated structural identifiability of model parameters, except parameter *k*_16_ which we set to one, and suggested practical identifiability of 11 out of 18 parameters (61%) (Figure S4). The best ranked model could reproduce the 4 min hyper-hypo-osmoshock Slt2 activation peak and it reproduced and predicted all other experimental data (Figs 3 and 4). Furthermore, all of the models with sensitizer were able to reproduce the 4 min Slt2 activation peak (Table 1).

### Model validation by dedicated follow-up experiments

**Figure 5:**
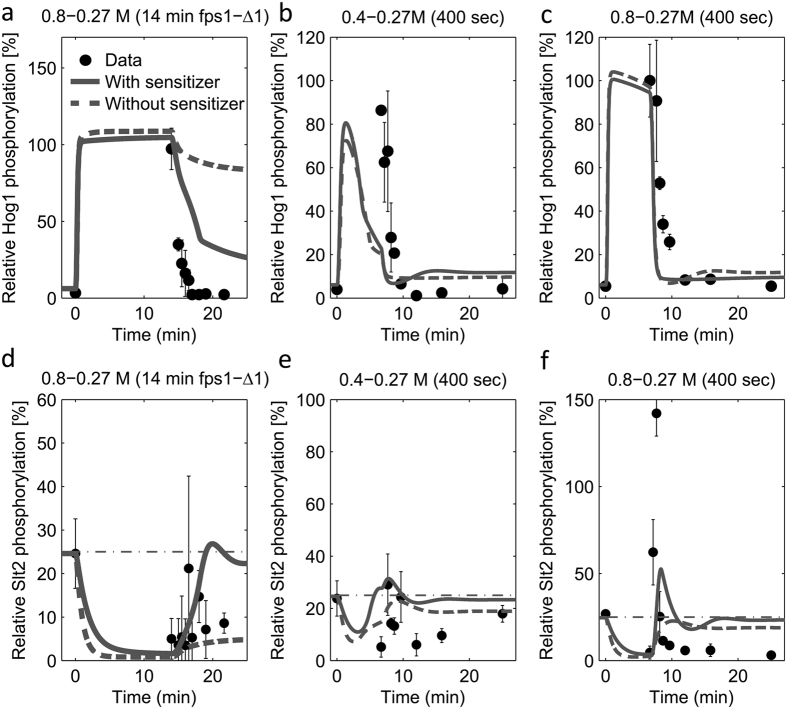
Experimental validation of sensitizer. Relative Hog1 and Slt2 phosphorylation data, used for further validation of model with sensitized negative feedback, are plotted versus time. Lines, except the vertical dash dot line, show model simulations and filled circles (•) show the experimental data (Mean ± SD (n = 3)). (**a/d**) Comparison between Hog1/Slt2 phosphorylation data and its simulation for 0.8 M sorbitol shock with subsequent dilution to 0.27 M sorbitol at 14′ in *fps1-Δ1* mutant (Fully open Fps1). (**b/e**) Comparison between Hog1/Slt2 phosphorylation data and its simulation for a sorbitol shock of 0.4 with subsequent dilution to 0.27 M at 400″, respectively. (**c/f**) Comparison between Hog1/Slt2 phosphorylation data and its simulation for 0.8M sorbitol shock with subsequent dilution to 0.27 M at 14′, respectively.

To further validate our model, especially the Slt2 sensitizer mechanism, we used the best ranked models with and without sensitizer, respectively, and designed follow-up experiments. Specifically, we designed experiments in which the predictions of the models with and without sensitizer would lead to significantly different predictions that we consecutively tested. One of these experiments was a hyper-hypo-osmotic shock (0.8–0.27 M), where the hypo-osmotic shock was applied after 14 minutes with a constitutively open Fps1 channel (*Fps1-Δ1* mutant). The models predicted that in the model with Slt2-sensitizer, Hog1/Slt2 activation would substantially drop/increase after hypo-shock, whereas in the model without sensitizer it would not. The data clearly supported the model with Slt2 sensitizer (Fig. 5a,d). Another experimental design leading to different model predictions was one in which we modulated the hyper-osmotic shock (0.4 M and 0.8 M) followed by hypo-osmotic shock (0.27 M) after 400 seconds. Simulations showed that the model with Slt2 sensitizer predicted a significant Slt2 activation above base levels (dashed line in Fig. 5d–f) in the 0.8–0.27 M hyper-hypo-osmotoic shock experiment, but not in the 0.4–0.27 M hyper-hypo-osmotic shock experiment, whereas the model without Slt2 sensitizer would predict no significant Slt2 activation in either experiment (Fig. 5b,c,e,f). Both models predicted a rapid Hog1 deactivcation upon hypo-osmotic shock. Although neither of the models could perfectly predict the data, again, the data clearly supported the model with Slt2 sensitizer (Fig. 5b,c,e,f).

### Autonomous activity of Hog1 and Slt2 MAPKS

A potential mode of crosstalk between the HOG and CWI pathways, that has been suggested, consists of a mutually inhibitory effect of the respective main kinases, i.e. Hog1 and Slt2, over each other’s phosphorylation/activation^19^,^20^. Our experimental data showed time-dependent activation of Slt2, variable Slt2 activation upon dilution at different times after hyper-osmotic stress, which could be the consequence of a combination of insufficient cell volume adaptation and an inhibitory effect of Hog1 on Slt2 activation.

The best ranked model supports the absence of direct crosstalk between Hog1 and Slt2 (Table 1). However, the second ranked model, which can be within the AICc cut off range (Table S3), supports an inhibitory effect of Hog1 on Slt2 phosphorylation (Table 1). This suggests that the corresponding hypothesis might be still valid. In any case, mutual crosstalk between the two pathways is predicted to have negligible significance, at least under the conditions considered in this study.

### Hog1 is the master regulator of Fps1

There is evidence suggesting that Slt2 mediates or enhances Fps1 opening^15^. Opposed to these findings, our selected model suggests negligible contribution of Slt2 in Fps1 opening, evidenced by the small estimated value of *k*_6_ (Table S8). However, the parameter identifiability analysis showed that the parameter *k*_6_ is practically non-identifiable (Figure S4). Thus, a lack of appropriate data can have led to an underestimation of Slt2’s influence on channel closure.

Garcia *et al*. showed strong Slt2 activation 60 minutes after 1.0 M of sorbitol shock^23^. They showed that this Slt2 activation is mainly due to the glycerol hyperaccumulation following sorbitol shock. Therefore, they postulated a significant contribution of Slt2 in opening the Fps1 channel leading to the release of hyperaccumulated glycerol.

To verify this result, we conducted an experiment with 1.0 M sorbitol shock and monitored Slt2 and Hog1 activation and cellular glycerol level. The experimental investigation suggested no marked Slt2 activation and no glycerol decrease 60 minutes following the sorbitol shock (Figure S5), supporting our model.

To further evaluate the hypothesized antagonistic effects of Hog1 and Slt2 on Fps1 regulation, we designed yet another experiment: if Slt2 played a significant role in opening Fps1 and glycerol release, Slt2 activation in cells having accumulated glycerol should lead to loss of glycerol, cell shrinkage and consequently Hog1 activation.

**Figure 6:**
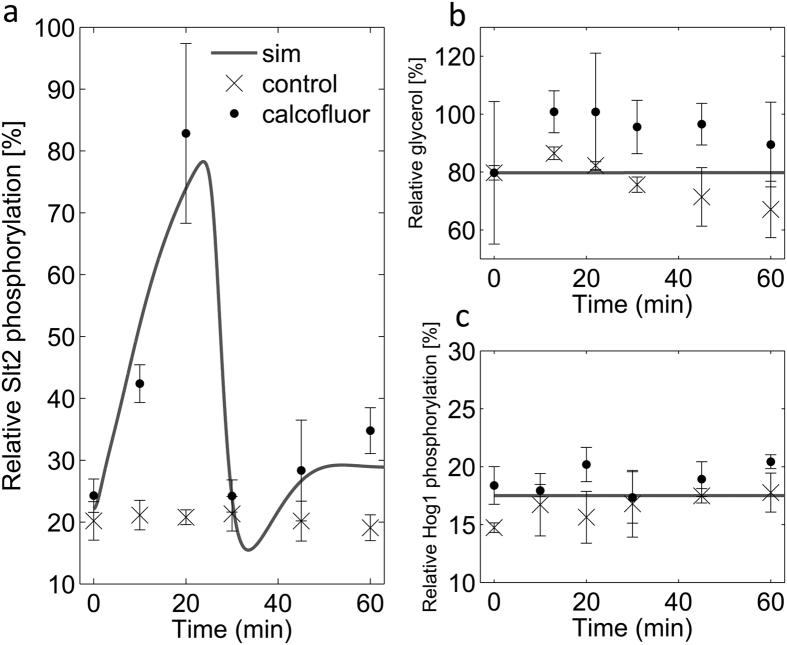
Experimental validation for no/weak Slt2 mediated Fps1 dephosphorylation. Relative Slt2 and Hog1 phosphorylation data, used for experimental validation of the model predictions, are plotted versus time. Solid lines show model simulations and cross marks (x) and filled circles (•) show the experimental data (Mean ± SD (n = 3)) for control and calcofluor exposed cells respectively. (**a**) The relative Slt2 phosphorylation/activation data under calcofluor exposure were used to calibrate the new module’s parameters. The model simulation shows Slt2 activation upon calcofluor exposure two hours following 0.8 M of sorbitol shock. (**b**) The normalized cellular glycerol level is plotted for control and calcofluor exposed cells at the time of calcofluor stress, *t* = 0, and 60 minutes after calcofluor exposure. The data do not show intracellular glycerol level decrease in calcofluor exposed cells. (**c**) Comparison of the relative Hog1 phosphorylation data with model simulation validates model prediction regarding no Hog1 activation upon calcofluor exposure two hours following 0.8 M of sorbitol shock.

The fluorescent dye calcofluor white has been shown to activate Slt2 by cell wall stress independently from osmotic stress^24^. Therefore, we used 0.11 μM (100 μg/mL) of calcofluor white for the activation of Slt2 two hours after 0.8 M of sorbitol shock (Fig. 6). The initial 0.8 M sorbitol shock increases the glycerol gradient between cell and medium prior to the calcofluor exposure amplifying the effects of any hypothetical glycerol leakage.

We monitored Slt2 and Hog1 phosphorylation in cells exposed to 0.11 μM of calcofluor white two hours after 0.8 M of sorbitol shock. Indeed, Slt2 was strongly induced upon calcofluor treatment (Fig. 6a). We used this Slt2 activation data to parameterize a new module which was designed and plugged in the selected model for activating the Slt2 upon calcofuor exposure (Figure S6, Supplementary Materials, Slt2 activating module). This module hypothesizes an integral negative feedback independent from osmotic pressures regulating the transient Slt2 activation after cell wall stress. The selected model simulations, augmented with the new Slt2 activating module, suggested no glycerol leakage and consequently no Hog1 activation upon calcofluor exposure. Indeed, this was confirmed by our experiments (Fig. 6b,c).

Taken together, our model as well as our follow-up experiments suggests that Slt2 activity plays no significant direct role in counteracting Hog1-mediated Fps1 closure under the studied conditions. The model rather implies that a constitutive mechanism counteracts Hog1, whose deactivation is consequently necessary and sufficient to open Fps1 in response to hypo-osmotic stress in wild type yeast. These results suggest a dominant role for Hog1 over Slt2 in Fps1 regulation.

## Discussion

We analyzed a series of time-dependent hyper-hypo-osmotic shock experiments using mathematical models to gain insight into the regulation of osmotic homeostasis in yeast. The models were kept as simple as possible and yet as complex as necessary to represent measured time series of Hog1 and Slt2 phosphorylation as well as intracellular glycerol concentration and cell volume. Despite their simplicity, the models were generally able to recapitulate our data. The best ranked model was able to predict the system behavior and was confirmed in dedicated follow-up experiments providing further support for some of the suggested hypotheses.

Apart from the volume regulation module, which has been developed and parameterized in earlier studies^1^,^3^,^13^, the pathways were represented by only two components; the signal component and the effector kinases Hog1 and Slt2 (Fig. 2). The signal was assumed to be volume dependent and to increase or decrease when a certain threshold was passed (Fig. 1d). The process of opening Fps1 and according loss of glycerol is quicker than production of new enzymes and glycerol. Therefore, adaption to hypo-osmotic shock condition is faster than adaptation to hyper-osmotic conditions. Concerning the HOG pathway this simple model could well explain and predict Hog1 activation for different conditions and mutants (Figures S1and S2). However, concerning the CWI pathway, this model could not explain an observed peak when external osmolarity was decreased after 4 min (Figure S1).

One possible explanation was that the volume threshold beyond which the CWI signal increases is not fixed, but is adaptive itself. Indeed, a new round of model development supported the hypothesis that upon volume shrinkage, the volume threshold above which the CWI pathway is activated also decreases. This suggests that when volume decreases the cell becomes more sensitive to sudden volume increase.

A recent study by Baltanas *et al*. suggested that Slt2 regulates opening of the Fps1 glycerol channel^15^. Our data did not support a model with a significant influence of Slt2 on Fps1 opening. When Hog1-induced closure of Fps1 was included in the model, rapid deactivation of Hog1 upon shift to hypo-osmotic condition was sufficient to re-open the channel provided a constitutive opening mechanism. A major role of Slt2 to further enhance or accelerate Fps1 opening was not required for rapid osmo-adaptation. This might be an artefact of the simplistic way we included Hog1/Slt2 control mechanisms in Fps1 regulation. In addition, Fps1 regulation parameters were practically non-identifiable, indicating that the data was not sufficient to train the model in this aspect. Notably, Lee *et al*.^8^ reported a mechanism that suits our model. They suggested that the Fps1 regulators Rgc1/2 maintain Fps1 in the open state in the absence of osmotic shock and that Hog1 activation induced the rapid eviction of Rgc1/2 from Fps1 and consequent channel closure. However, they did not study Fps1 regulation under hypo-osmotic conditions. A recent study, suggests another Hog1 independent meachanism of Fps1 opening, i.e. phophorylation by the TORC2-dependent protein kinase Ypk1^25^. Fps1 phosphorylation by Ypk1 is blocked upon hyper-osmotic shock, leading to Fps1 closure. Whether this mechanism can also lead to further Fps1 opening upon hypo-osmotic conditions remains to be investigated.

Here, we studied the contributions of Hog1 and Slt2 in Fps1 regulation by activating the Slt2 kinase using 0.11 μM of the fluorescent dye calcofluor white, following 0.8 M of sorbitol shock. This leads to Slt2 activation in cells which have already accumulated significant amounts of glycerol. If Slt2 played a significant role in opening the Fps1 channel, the model predicted Hog1 activation due to the loss of glycerol and, of course, the reduced intracellular osmotic pressure. However, we oberserved neither loss of glycerol nor Hog1 activation, supporting our model and the negligible role of Slt2 in opening Fps1 under these conditions (Fig. 6). Note that this experiments mimics an experimental setup of Baltanas *et al*.^15^, only that Baltanas *et al*. let cells adapt to high osmolartity over night instead of two hours, and they stimulated with pheromone instead of calcofuor to stimulate Slt2 activcation. Interestingly, Baltanas *et al*. came to opposite conclusions, suggesting that Slt2 does lead to glycerol release and corresponding Hog1 activation and glycerol re-production, which we do not observe. However, Baltanas *et al*. did not measure initial glycerol loss in their setup, but only glycerol re-production. Earlier studies suggested that after long-term adaptation to hyper-osmotic conditions, glycerol might be replaced by other osmolytes, e.g. trehalose^6^. Taking that into account, the results of Baltanas *et al*. can also suggest that Slt2 activation upon pheromone stimulus leads to loss of other osmolytes than glycerol, but still activating a Hog1 response and glycerol-reproduction. This in turn suggests that Slt2 might still be involved in osmoregulation, however, not through the glycerol channel Fps1.

Note that we avoided genetic manipulations, but rather designed experimental conditions to separate the effects of the two pathways. Genetic manipulations would probably also change other processes influenced by Hog1 and Slt2, like cell cycle arrest or cell wall modifications. These processes are not explicitly considered here, but are assumed to have a constant yet condition-independent effect. Genetic manipulations would change this effect and, therefore, the parameters of our model. Consequentlty, our model would no longer be suitable to simulated such conditions (Figure S7).

Another hypothesis which can explain Baltanas *et al*. observations is that the pheromone stimulation might activate the newly discovered Ypk1 mediated Fps1 opening leading to a glycerol loss and Hog1 activation while calcofluor exposure only activated the Slt2 pathway. This hypothesis would reject or disregard Hog1 activation via Slt2 mediated osmolyte loss in any form unless pheromone activation of TORC2/Ypk1 is somehow dependent on Slt2 activity.

Taken together, our results suggest that Hog1 dominates Fps1 regulation and short-term osmo-adaptation both under hyper- as well as hypo-osmotic conditions. However, we do not exclude the possibility of Slt2 mediated Fps1 regulation in general, since our study is confined to conditions of osmotic pressure changes. In addition, Slt2 activation is mediated in cells with elevated Hog1 phosphorylation due to the sorbitol shock before calcofluor exposure. Therefore, the corresponding experimental condition may not reflect Fps1 regulation dynamics during weak/no Hog1 activation. Moreover, Slt2 might be implicated in the regulation of other osmolytes than glycerol.

Although previous studies suggested a crosstalk between HOG and CWI pathways on the kinase level upon zymolase exposure^14^,^19^,^20^, our data supported a model without crosstalk. This is further supported by the fact that the second ranked model, with inhibitory effect of Hog1 on Slt2 activation, showed a negligible crosstalk between those pathways. This lack of support for crosstalk on the kinase level might explain why no significant crosstalk on the Fps1 level was observed as well.

This study presents a new perspective on cellular osmoregulation. Our model suggests that the precise dynamical regulation of only one component, the positive regulator of osmotic pressure Hog1, guarantees a proper osmotic stress response to both hyper and hypo-osmotic challenges. Alternative or antagonisitc Hog1-independent mechanism of Fps1 closure has been reported by Muir *et al*., but it is not as efficient as Hog1-mediated closure as studied under hyper-osmotic shock condition^26^. However, why Hog1 dominates the newly discovered Ypk1 pathways remains to be investigated.

## Materials and Methods

### Methods

#### Hypo osmotic shocks induction

One way to establish the hypo-osmotic condition is to dilute the medium with pure water. However, this may reduce sugar concentrations to values stimulating a starvation response potentially making it difficult to separate the nutritional from the osmotic response. Therefore, we first applied a hyper-osmotic shock with sorbitol (0.8 M), and then decreased the sorbitol concentration again (0.27 M) by dilution with standard medium (Fig. 1b,c) keeping the nutrient concentrations approximately constant.

#### Ensemble modelling of the hyper-hypo-osmotic shock response

We assumed that both the HOG and the CWI pathway respond to the difference between a given steady state volume and the actual volume, but in opposite directions (Fig. 1d). Both pathways have an initial basal activity, which, beyond a certain volume threshold (*k*_0_/*k*_2_ in Fig. 1d), linearly increases when the volume drops/increases below/above base levels for the HOG (solid line in Fig. 1d) and the CWI pathway (dashed line in Fig. 1d), respectively. This behavior can be modeled by simple generic signaling modules, which have a similar structure for both pathways (Fig. 1d). The advantage of such modules is that they avoid discontinuous rule-based modelling, thereby allowing for a wider range of steady state analysis methods. For the HOG pathway, such a signaling module has been proven to be useful^27^.

Here, we adopted the generic structure employed in Schaber *et al*.^27^, which we extended by a simple established and already parameterized biophysical module^1^,^6^,^13^. In Fig. 2 we depict the wiring schemes of the model family representing different hypotheses about possible crosstalk mechanisms between the HOG and the CWI pathway (dashed lines a and b in Fig. 2) that are motivated by literature^7^,^8^,^14^,^15^,^19^,^20^. Models were generated based on the principle of parsimony, i.e., as simple as possible and as complex as necessary to explain data and answer our research questions. The basic structure of the HOG and the CWI pathway model is similar. The core of the model is constituted by the two effector kinases Hog1 and Slt2, which are the main representatives and experimental read-outs of both pathways. Both kinases are activated (*v*_3_ and *v*_12_) by a generic signal (*HOGSignal* and *CWISignal*, respectively, Fig. 2) and constitutively deactivated (*v*_*4*_ and *v*_*13*_). The total amount of both kinases is assumed to be constant, i.e. they are only transformed between an inactive and an active state. The active state of Hog1 leads to an increase in internal glycerol production (*v*_*7*_), whose leakage into the medium (*v*_8_) is controlled by Fps1. The difference between the internal osmolarity, represented by internal glycerol (*Gly*_in_), and the external osmolarity (*Osm*_ex_), which is mainly influenced by external sorbitol concentration (*Sorbitol*), controls the volume (for details please refer to the Supplementary Material). The volume, in turn, regulates Hog1 and Slt2 activity through the generic signals *HOGSignal* and *CWISignal*, respectively, down-regulating the HOG signal (*v*_2_) and stimulating the CWI signal (*v*_9_), respectively. Moreover, the volume independently affects the concentrations of all components in the cytoplasm (light gray components in Fig. 2). For simplicity we do not distinguish between cytoplasm and nucleus. Active Hog1 has been shown to lead to the phosphorylation of positive regulators of the Fps1 channel, Rgc1/2, and closure of the channel (*v*_5_)^7^,^8^. Active Slt2 has been hypothesized to regulate Fps1 opening (*v*_6_) (alongside Slt2 independent Fps1 opening *v*_6basal_). Model options include different crosstalk combinations at the kinase level, where the active forms of both kinases inhibits activation of the other kinase (dashed lines a and b in Fig. 2). At a later stage, we included a sensitizer in the Slt2 pathway, which its rational and functioning is described in the result section. All mathematical models are explained in detail in the Supplementary Materials (Mathematical Models, Tables S4–S8).

#### Model Inference and Selection Strategy

The main source for data were time series of double-phosphorylated Hog1 and Slt2, cell volume and intracellular glycerol levels for different hyper-hypo-osmotic shock regimes. We split the data into two sets, one for parameterization and one for validation. For parameterization, we used the single 0.8M sorbitol hyper-osmotic shock experiment and 0.8–0.27 M sorbitol dilution experiments with dilution at 4, 14 and 30 min, the volume data for 0.8 M sorbitol hyper-osmotic shock and also a Hog1 inhibition experiment that was used in earlier studies^15^,^28^ (Fig. 3). For model validation, we used the 0.8–0.27 M sorbitol dilution experiments with dilution at 45 s, 90 s and 45 min, as well as the glycerol data for 0.8 M sorbitol hyper-osmotic shock (Fig. 4). Note that the validation experiments featured dilutions before and after those experiments used for parameterization. This way extrapolating properties of the model were tested. Both the squared residuals of the fitted as well the predicted data entered the final weighted sum of squared residuals (*wSSR*) that was used to rank the models according to the Akaike Information Criterion corrected for small samples (*AICc*)^21^. However, we further confirmed the model selection by repeating model ranking using all experimental data for parameter estimation. For details on the fitting and ranking procedure, please refer to the Supplementary Material. The selected model can be found in the online Supplementary Materials both in COPASI and SBML formats as well as in the BioModels database^29^ (access identifier MODEL1606100000). Moreover, instructions regarding the simulation of experimental data using the selected model are provided in the Supplementary Material (Simulation Instructions).

### Experimental methods

#### Yeast strains, media, plasmids and sampling.

Wild type cells in the W303 strain background and W303 cells transformed with the relevant plasmids. Cells were grown in Yeast Nitrogen Base medium (1x Difco™ YNB base, 1x Formedium™ Complete Supplement Mixture, 5.0 g/L ammonium sulfate, 20 g/L glucose) to exponential phase. Plasmids used were YEplac195 and YEplac195-*fps1∆1* for experiments with constitutively open Fps1^22^. For all sampling cells were grown to mid-log phase in YNB medium. After addition of stress agent to cultures samples were taken at indicated time points with zero samples taken before addition of stressor. For hyper-osmotic stress sorbitol was added to the medium to a final concentration of 0.8 M or 1.0 M sorbitol depending on the experiment, for initial model validation 0.4 M NaCl was also used for hyper-osmotic stress. For the calcofluor treatment 0.11 μM (100 μg/mL) was added to the medium.

#### Single cell analysis of cell volume

Cell volume was monitored using a microfluidic system with three inlet channels as described previously^30^,^31^. Images of approximately 60 cells were taken sequentially every 30 s for 300 s, every 60 s for 600 s, every 120 s for 600 s and every 600 s for 1200 s thus yielding a total experiment period of 45 min. The images were analyzed using the CellStress software^32^.

#### Protein extraction and Western blotting

800 μL samples were taken at indicated timepoints. To fix cells samples were immediately added to 150 μL fixation buffer (80% Tri-chloro-acetic acid, 100 μg/mL cycloheximide) and put on ice. Cells were then collected via centrifugation. The pellet was resuspended in 1 mL wash buffer (600 mM Tris-HCl, 40% aceton) centrifuged and the resulting pellet was left to let residual acetone evaporate. To the pellet, 30 μL glassbeads and 60ul of extraction buffer (90 mM Tris-HCl [pH 6.8], 200 mM dithiothreitol, 3% SDS, 13% glycerol, 2% mercaptoethanol, 10 mM NaF, 0.1 mM Sodium orthovanadate, protease inhibitor [complete EDTA-free protease inhibitor cocktail tablets; Roche], 0.005% Bromophenol Blue) was added. Cell suspensions were vortexed for 2 min, boiled for 6 min and then centrifuged at 13,000 × g for 10 min to obtain pure protein extracts. Extracts were stored at −80 °C. 15 μL of protein was separated on an SDS (10%) polyacrylamide gel and blotted onto nitrocellulose membranes (Hybond^TM^ ECL^TM^, Amersham). Membranes were blocked with Odyssey blocking buffer PBS (LI-COR Biosciences) for 1 h and incubated overnight at 4 °C with the anti-phospho-p38 MAPK primary antibody (Thr180/Tyr182; Cell Signaling), anti-phospho-p42/44 MAPK primary antibody (Thr202/Tyr204; Cell Signaling) and total anti-Hog1 (yC-20, Santa Cruz), diluted 1:1000 in Odyssey blocking buffer (0.1% Tween 20). After washing (4 × 5 min in TBS-T), membranes were incubated with IRDye 680 donkey anti rabbit and IRDye 800 donkey anti goat (LI-COR Biosciences) secondary antibodies diluted 1:15,000 in Odyssey blocking buffer for 1 h at room temperature. The washing step was repeated before scanning (Odyssey scanner, LI-COR Biosciences). Westerns were quantified using Image Studio Lite (LI-COR Biosciences).

### Measurement of intracellular glycerol

Cells were harvested from 1 mL cell culture samples by centrifugation, the supernatant was carefully removed via pipetting and the samples frozen in liquid nitrogen. Samples were later resuspended in 1 mL of water, boiled at 100 °C for 10 min, centrifuged and 800 μL of the resulting supernatants were stored at −20 °C. OD600 was determined at all-time points. Glycerol concentration was determined using a commercial kit (r-biopharm Cat. No 10148270035). Reaction was scaled down 12 times to a final reaction volume of 250 μL. Measurements were performed in a 96-well plate using a Polar Star Omega plate reader (BMG Labtech).

## Additional Information

**How to cite this article**: Talemi, S. R. *et al*. Systems Level Analysis of the Yeast Osmo-Stat. *Sci. Rep.* **6**, 30950; doi: 10.1038/srep30950 (2016).

## Supplementary Material

Supplementary Information

Supplementary Information

## Figures and Tables

**Table 1:**
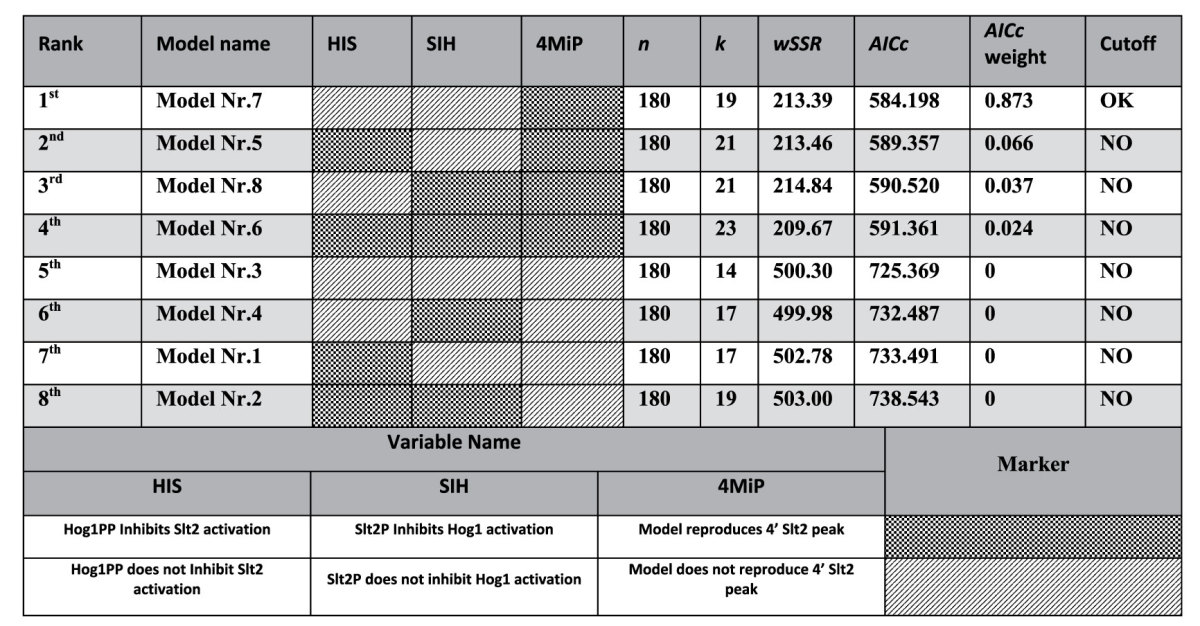
Models are ranked according to Akaike Information Criterion corrected for small sample size (*AICc*).

The data from NoHYPOS, 45″, 90″, 4′ (0.8 M sorbitol to 0.4 and 0.5 M dilutions respectively) and 45′ dilution experiments were not used for parameter estimation (*wSSR*). All models with sensitizer component were ranked in top 4 and were able to fit 4′ Slt2 activation peak (4MiP). The best ranked model shows no cross talk between Hog1 and Slt2 (HIS and SIH). Abbreviations: *n*: number of data points, *k*: number of parameters, *wSSR*: weighted sum of squared residuals, *AICc*: Akaike Information Criterion corrected for small sample size, *AICw*: Akaike weights.
