# Supplementary material for: Systems Level Analysis of the Yeast Osmo-Stat
Source: Sci Rep. 2016 Aug 12;6:30950. doi: 10.1038/srep30950 (PMC4981887; doi:10.1038/srep30950)
Supplement: Supplementary Information [file srep30950-s1.pdf]

Systems Level Analysis of the Yeast Osmo-Stat

Supplementary Information

Soheil Rastgou Talemi[1,2#], Carl-Fredrik Tiger[3#], Mikael Andersson[3], Roja Babazadeh[3], Niek Welkenhuysen[3], Edda Klipp[4], Stefan Hohmann[3,5,*], Jörg Schaber[1,*]

[1]Institut für Experimentelle Innere Medizin, Medizinische Fakultät , Otto-von-Guericke-Universität, Magdeburg, Germany

[2]Division of Theoretical Systems Biology, German Cancer Research Center (DKFZ), Heidelberg, Germany

[3]Department of Chemistry and Molecular Biology, University of Gothenburg, SE-405 30 Göteborg, Sweden

[4]Theoretical Biophysics, Humboldt Universität zu Berlin, Berlin, Germany

[5]Department of Biology and Biological Engineering, Chalmers University of Technology, SE-412 96 Göteborg, Sweden

[#]These authors contributed equally

* To whom correspondence should be addressed:

Jörg Schaber; Phone: +49 391 67 14453; Fax: +49 391 67 13312;

Email: j.schaber@web.de

Stefan Hohmann; Phone: +46 31 3608488; Fax: +46 31 786 2599

E-mail: stefan.hohmann@chalmers.se

Contents

Data Scaling 3

Hog1 phosphorylation data 3

Slt2 phosphorylation data 3

Volume data 3

Glycerol data 3

Methods 3

Parameter Estimation 3

Model Selection 2

Identifiability Analysis 2

Mathematical Models 2

Modified Model Changes 4

Calcofluor mediated Slt2 activating module 4

Supplementary Figures 8

Figure S1: 10

Figure S2: 11

Figure S3: 12

Figure S4: 13

Figure S5: 14

Figure S6: 15

Figure S7: 15

Supplementary Tables 16

Table S1: 16

Table S2: 17

Table S3: 18

Table S4: 19

Table S5: 20

Table S6: 22

Table S7: 25

Table S8: 28

Supplementary References: 31

Data Scaling

Hog1 phosphorylation data

We scaled the Hog1 phosphorylation data for model calibration. Hog1 phosphorylation levels were scaled to the maximum phosphorylated Hog1 after 0.8 M sorbitol shock, assuming that this value is the maximum Hog1 phosphorylation level.

Slt2 phosphorylation data

We scaled the Slt2 phosphorylation data for model calibration. Slt2 phosphorylation levels were scaled to the maximum phosphorylated Slt2 upon 0.8 M hyper-osmotic shock followed by dilution to 0.27 M of sorbitol 30 minutes after. For the validation data we assumed that the average initial Slt2 phosphorylation level is 25% of the maximum explained in previous condition.

Volume data

We scaled the volume measurements to the cell volume prior to 0.8 M sorbitol shock.

Glycerol data

We scaled the glycerol measurements to the normalized measured glycerol 45 minutes after 0.8 M sorbitol shock (Eqn. 1).

$$\text{Relative glycerol level} = \frac{\frac{[Glycerol]}{Optica\ Density\ (OD)} * V_{Rel}}{\left[\frac{[Glycerol]}{Optica\ Density\ (OD)} * V_{Rel}\right]_{45min}} \times 100 \quad (1)$$

V_{Rel}=relative volume.

Methods

Parameter Estimation

Model parameters estimation was done using COPASI (version: 4.15) [1]. The Evolutionary Programming method was used to estimate model parameters. The weighted Sum of Squared Residuals (*wSSR*) was used as objective function (Eqn. 2).

$$wSSR = \sum_{i=1}^{m} w_i \sum_{j=1}^{n} (\hat{y}_{i,j} - y_{i,j})^2 \quad (2)$$

with i=1,…,m as the number of experiments, and j=1,…,n as the data pointed for experiment i. w_i represents the respective weight of experiment i, set to the inverse of the average of the respective time series. $\hat{y}_{i,j}$ is the simulated value for data point number j within experiment i and $y_{i,j}$ is the measured data point j within experiment i. We used the 0.8-0.27 M sorbitol hyper-hypo-shock experiments with hypo-shock at 4', 14' and 30' as well as the volume data for 0.8 M sorbitol hyper-shock to fit the model parameters.

Model Selection

In order to select the most parsimonious mathematical model, which best approximates the data, we used the Akaike Information Criterion corrected for small sample sizes (AIC_c) (Eqn. 3). AIC_c is an information theoretic approach for model selection, based on Kullback-Leibler (K-L) concept of information loss when using a model to approximate full truth. The full truth includes an infinite number of parameters, which determine the systems output [3].

$$AIC_c = 2k + n\left(ln\left(\frac{2\pi \cdot wSSR}{n}\right) + 1\right) + \frac{2k(k+1)}{n-k-1} \quad (3)$$

where k, n and $wSSR$ represent number of parameters, number of data points and the weighted sum of squared residuals, respectively. Finally, models were ranked according to AIC_c, where the model with the minimum AIC_c score was ranked first. The K-L confidence set comprised of all models for which their likelihood relative to the estimated K-L best model likelihood, be ≈ 1/8 [3].

In order to select and compare the best approximating model(s) we calculated the Akaike weights (*AICw*) (Formula 4) [3].

$$AICw_i = \frac{e^{-\frac{1}{2}\Delta_i}}{\sum_{r=1}^{R} e^{-\frac{1}{2}\Delta_r}} \quad (4)$$

where $\Delta_i = AIC_i - AIC_{min}$, with AIC_i being the *AICc* for model i, i=1, …, R according to ranking and AIC_{min} the minimal *AICc*. The *AICws* can be considered as the weight of evidence in favour of a model given as a number between 0 and 1, i.e. the higher the weight, the closer the model is to the hypothetical true model [3]. We considered those models as the best approximating for which the relative value of Akaike weight is > *1/8*. The relative Akaike weight is the ratio of the models Akaike weight to the Best ranked model Akaike weight (Formula 5) [2,3].

$$\frac{AICw_i}{AICw_{max}} = \exp\left(-{}^{1}\!/_{2}\,\Delta_i\right) \quad (5)$$

Identifiability Analysis

We conducted profile likelihood based identifiability analysis [4] using COPASI as explained in the literature [5]. This method identifies structural as well as practical identifiability. Models with structural non-identifiability cannot be trained by the data. The non-identifiable model parameters cannot be trained by the data.

Mathematical Models

Three components were implemented differently leading to different candidate models. Each of these three components can adopt two possible setups. Thus, 8 different combinations were generated. The alternative model formulations are

indicated by dashed components in Fig. 2. For a better overview we shortlist the components and their setups:

A) Activate Hog1 inhibits Slt2 activation

Two sets of models were designed based on inhibitory effect of the Hog1 on Slt2 activation upon hypo-osmotic shock.

I. Hog1 inhibits Slt2 activation.
II. Hog1 does not inhibit Slt2 activation.

B) Active Slt2 inhibits Hog1 activation

Two sets of models were designed based on inhibitory effect of the Slt2 on Hog1 activity upon hypo-osmotic shock.

I. Slt2 inhibits Hog1activity.
II. Slt2 does not inhibit Hog1 activity.

C) Sensitized negative feedback on *CWISignal* degradation

Two sets of models were designed based on sensitized/not-sensitized regulation of *CWISignal* activation threshold:

I. There is a sensitized negative feedback from *CWISignal* to its degradation rate
II. There is no sensitized negative feedback from *CWISignal* to its degradation rate

Mathematical formulation of models is explained in Tables S4-S8. The order of mathematical details in these tables is explained below:

Table S4:

This table lists ordinary differential equations of the master model.

Table S5:

This table lists the rate laws for the reactions from Table S4 and details the differences between the model alternatives.

Table S6:

This table lists the state variables and their initial conditions for the selected model. As models are initially set to steady state, some initial conditions are calculated from estimated/set ones. The latter are listed in Table S8.

Table S7:

This table lists auxiliary variables and physical quantities including volume, molar weight and cell surface calculation.

Table S8:

This table lists all estimated parameters including rate constants and initial conditions for the selected model.

Modified Model Changes

In order to reproduce the 4′ Slt2 phosphorylation peak we increased the glycerol production approximately by a factor of 2.

$$k_7 = 935.301 \rightarrow 1870\ (\frac{\mu mol}{fl * Sec})$$

Calcofluor mediated Slt2 activating module

No model inside the models ensemble was designed such that can respond to the presence of the calcofluor in the medium. Therefore, we designed a new mathematical module that is able to activate the Slt2 upon calcofluor exposure. The new mathematical module is comprised of 5 species, namely *Calcofluor*, *CALSignal*, *Degrader, Slt2 and Slt2P* (Figure S7a). The three new species *Calcofluor*, *CALSignal and Degrader*, represent the calcofluor white; the signal which activates Slt2; and a component which degrades the Slt2 activating signal, respectively. The corresponding module was then plugged in the model main model (Figure S7b). The new mathematical module parameters were estimated from Slt2 activation dynamics upon 0.11 µM of calcofluor white, two hours after 0.8 M of sorbitol shock. No parameter from the selected model was dedicated for parameter estimation for reproducing the corresponding experimental result. The mathematical formulation of this mathematical module, its parameter values and the components initial concentrations are explained below:

Rate laws:

$$v_a = k_a \cdot [Calcofluor],$$

$$v_b = k_b \cdot [CALSignal] \cdot [Slt2],$$

$$v_c = \frac{V_{\max\_c} \cdot [Slt2P]}{(K_{m\_c} + [Slt2P])},$$

$$v_d = \frac{V_{\max\_d} \cdot [CALSignal] \cdot [Degrader]^h}{({S_{half}}^h + [Degrader]^h)},$$

$$v_e = \frac{V_{\max\_e} \cdot Degrader}{(K_{m\_e} + Degrader)}.$$

Initial concentrations:

$$[Calcofluor] = \begin{cases} 0 & time < 2hours \\ 1 * (1 - e^{-(\frac{time - 2hours}{5})}) & else \end{cases},$$

$$[CALSignal]|_{t=0} = 0,$$

$$[Degrader]|_{t=0} = 0,$$

$[Slt2]|_{t=0}$ = see Table S6.

$[Slt2P]|_{t=0}$ = see Table S6.

Estimated parameters:

$k_a = 0.00133916$ s^{-1},

$k_b = 0.00860898$ µM^{-1}· s^{-1},

$V_{\max\_c} = 5.29577$ µM· s^{-1},

$K_{m_c} = 1384.04$ µM,

$V_{\max\_d} = 27583.6$ s^{-1},

$S_{half} = 13.0308$ µM,

$h = 16.4149$,

$V_{\max\_e} = 0.0142624$ µM^{-1}· s^{-1},

$K_{\mathrm{m}\_e} = 61.527$ µM^{-1}· s^{-1}.

Simulation Instructions

All models were implemented and calibrated using COPASI software, which allows for exporting models in Systems Biology Markup Language (SBML). The selected model is available as supplementary files both in COPASI and SBML (level2, version 4) formats. The selected model can be found in the online Supplementary Materials both in COPASI and SBML formats as well as in the BioModels database[28] (access identifier MODEL1604100004). Different experimental conditions can be simulated using the model and COPASI software. The 0.8 M sorbitol stress response is the simplest experiment that can be simulated using the selected model. To this end, after opening the ".cps" file by COPASI software, extracellular sorbitol concentration should be set to 0.8 M of sorbitol by setting model's parameter s1 to 800000 (µmol). The parameter s1 can be found under Model>Biochemical>Global Quantiles tabs in the COPASI file.

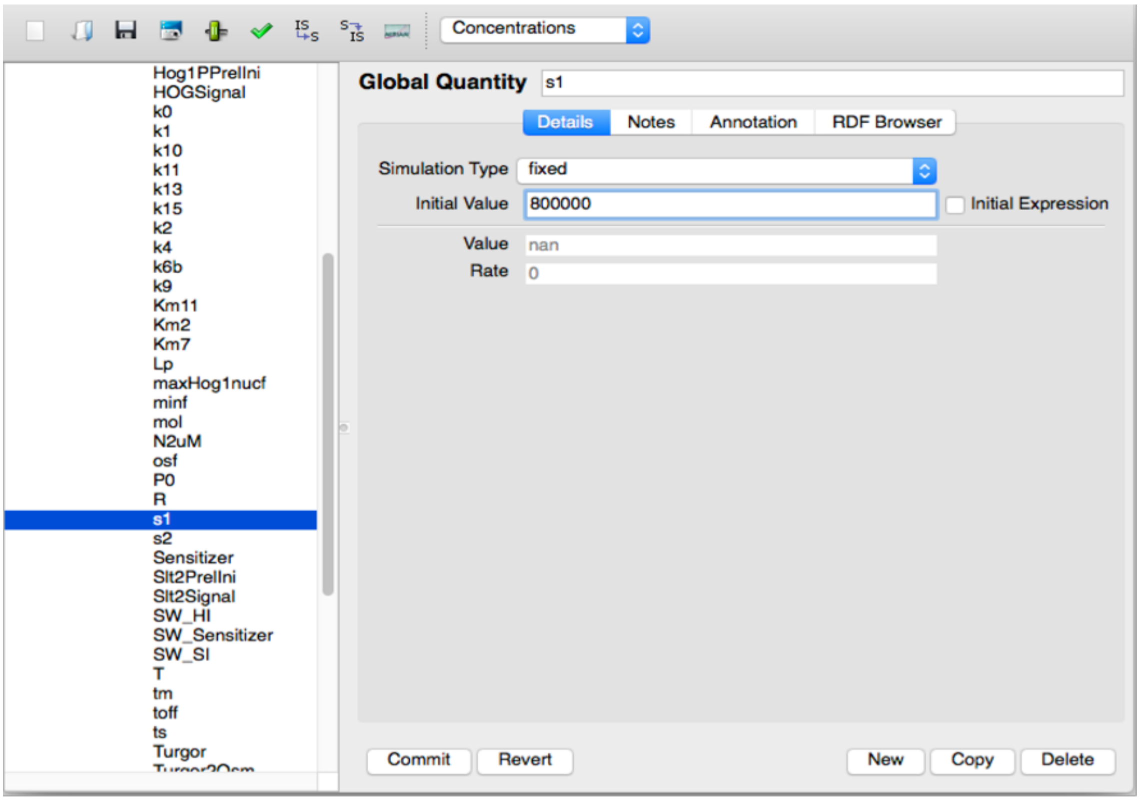

Moreover, further explanations regarding s1 parameter can be found in the supplementary table S7 and in the COPASI file under Model>Biochemical>Global Quantiles> cen parameter.

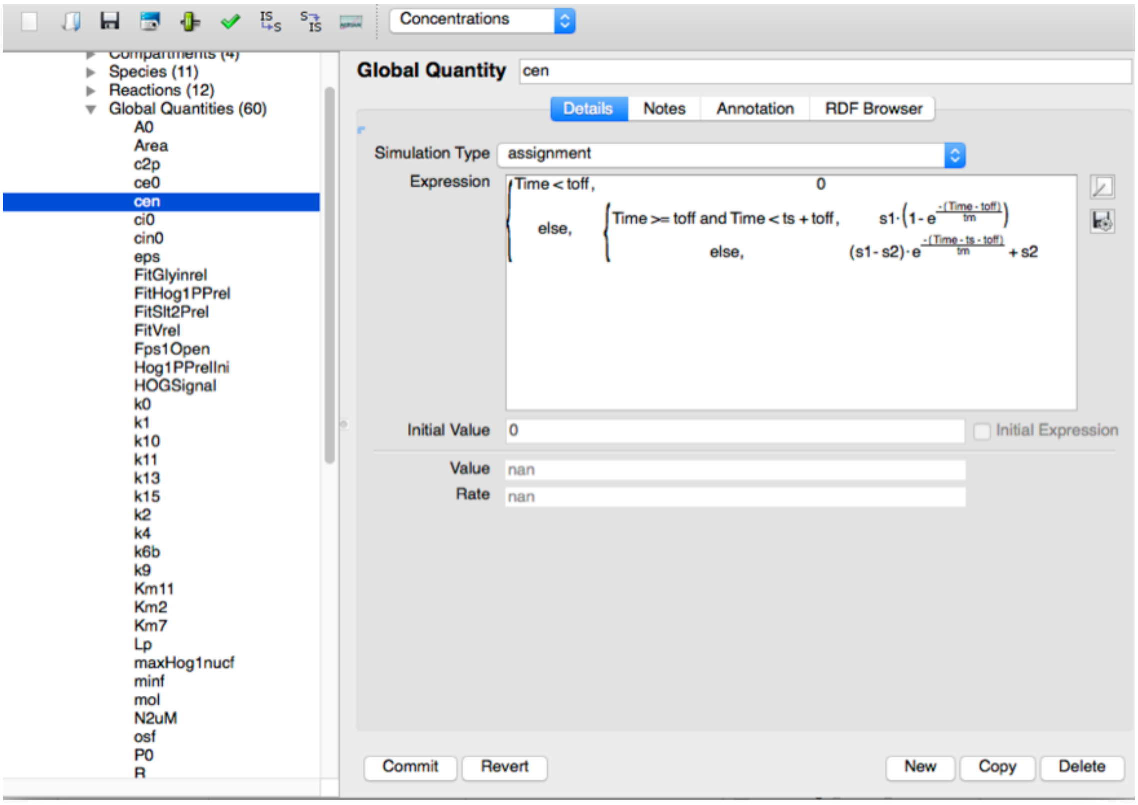

After setting this parameter, the 0.8 M sorbitol stress can be simulated using COPASI. Simulations can be conducted using Time Course task in COPASI, Tasks> Time Course. As a sample the simulation of the relative amounts of the Hog1PP and Slt2PP are shown in the graph. The red and blue curves show Hog1PP and Slt2PP respectively. It should be noted that the ordinate label in the simulation graph is automatically adopted by COPASI, which should be corrected for different simulations when reporting the plot.

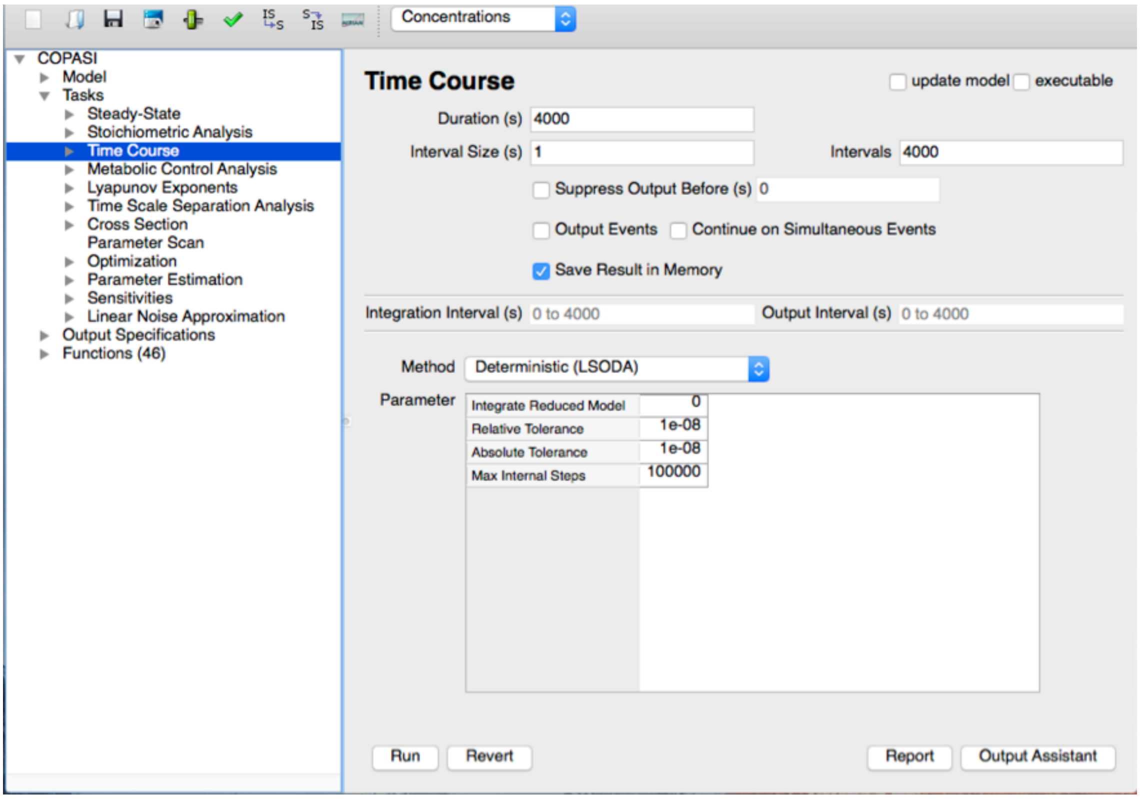

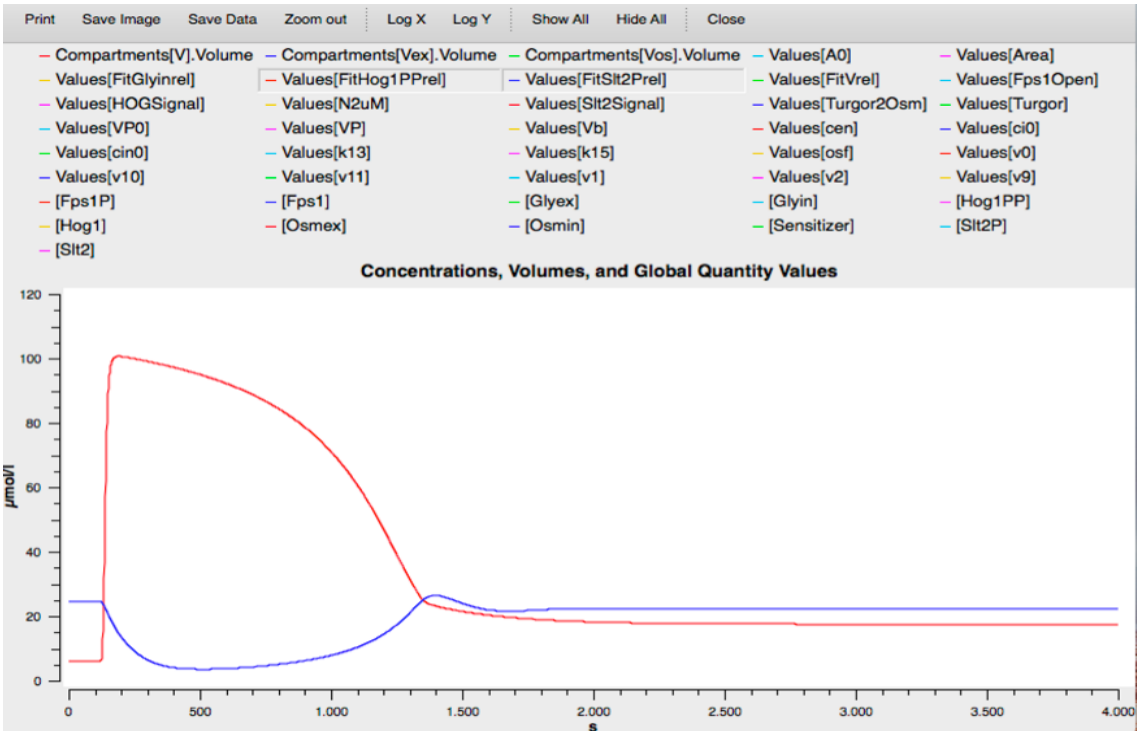

The hyper-hypo-osmotic stress experiment, 0.8 M sorbitol stress followed by dilution to x M of sorbitol can easily be simulated by COPASI. Additional to the initial sorbitol stress parameter, s1 = 800000, two other parameters should be adjusted. The first parameter regulates the time between the hyperosmotic stress and the dilution, ts (s). The second one, namely, s2, regulates the final external sorbitol concentration that we want to reach (supplementary table S7). For example, to impose a hyper-hypo-osmotic stress with initial sorbitol concentration of 0.8 M and the dilution to 0.27 M of sorbitol 14 min following the initial hyperosmotic stress, one needs to set the above mentiond parameters as below:

s1= 800000 (µmol), s2 = 270000 (µmol), ts = 840 (s).

Following this setting the model can be simulated using time course task as explained earlier. The red and blue curves show Hog1PP and Slt2PP respectively.

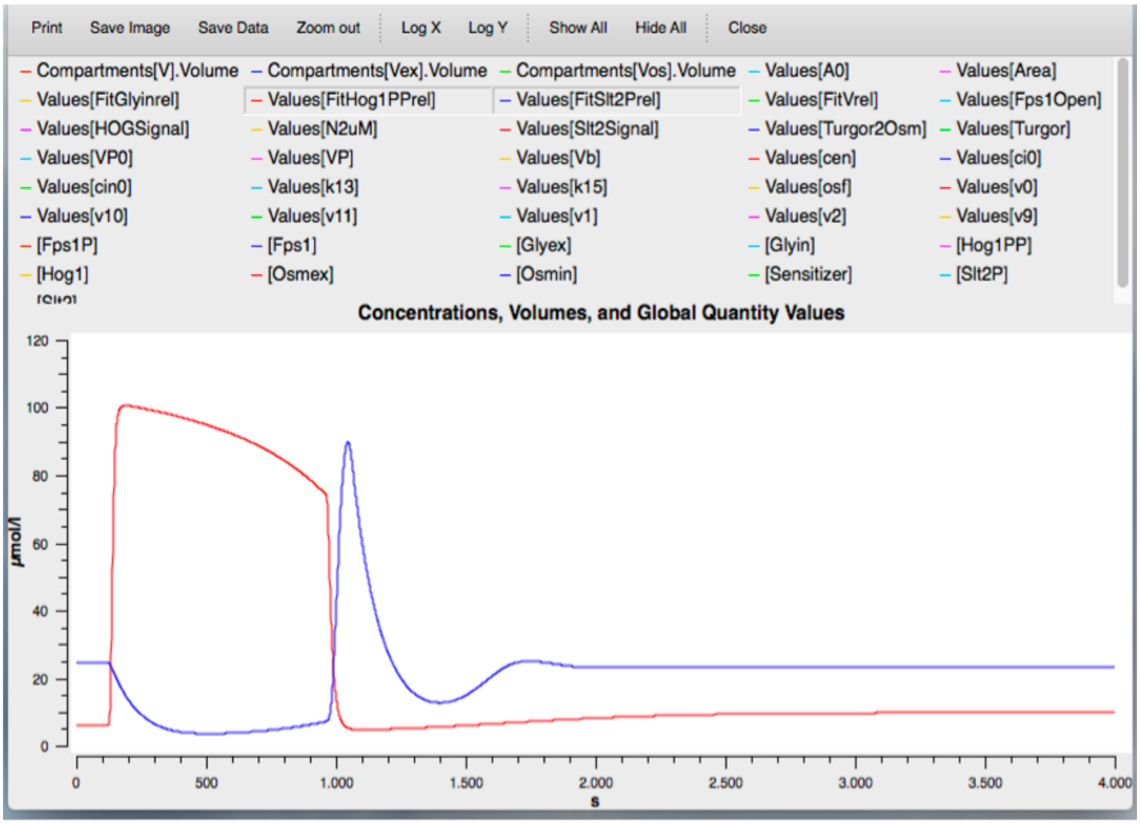
Print Save Image Save Data Zoom out Log X Log Y Show All Hide All Close
Compartments[V].Volume
Compartments[Vex].Volume
Compartments[Vos].Volume
Values[A0]
Values[Area]
Values[FitGlyinrel]
Values[FitHog1PPrel]
Values[FitSlt2Prel]
Values[FitVrel]
Values[Fps1Open]
Values[HOGSignal]
Values[N2uM]
Values[Slt2Signal]
Values[Turgor2Osm]
Values[Turgor]
Values[VP0]
Values[VP]
Values[Vb]
Values[cen]
Values[ci0]
Values[cin0]
Values[k13]
Values[k15]
Values[osf]
Values[v0]
Values[v10]
Values[v11]
Values[v1]
Values[v2]
Values[v9]
[Fps1P]
[Fps1]
[Glyex]
[Glyin]
[Hog1PP]
[Hog1]
[Osmex]
[Osmin]
[Sensitizer]
[Slt2P]
Concentrations, Volumes, and Global Quantity Values
µmol/l
120
100
80
60
40
20
0
0
500
1.000
1.500
2.000
2.500
3.000
3.500
4.000
s

Supplementary Figures

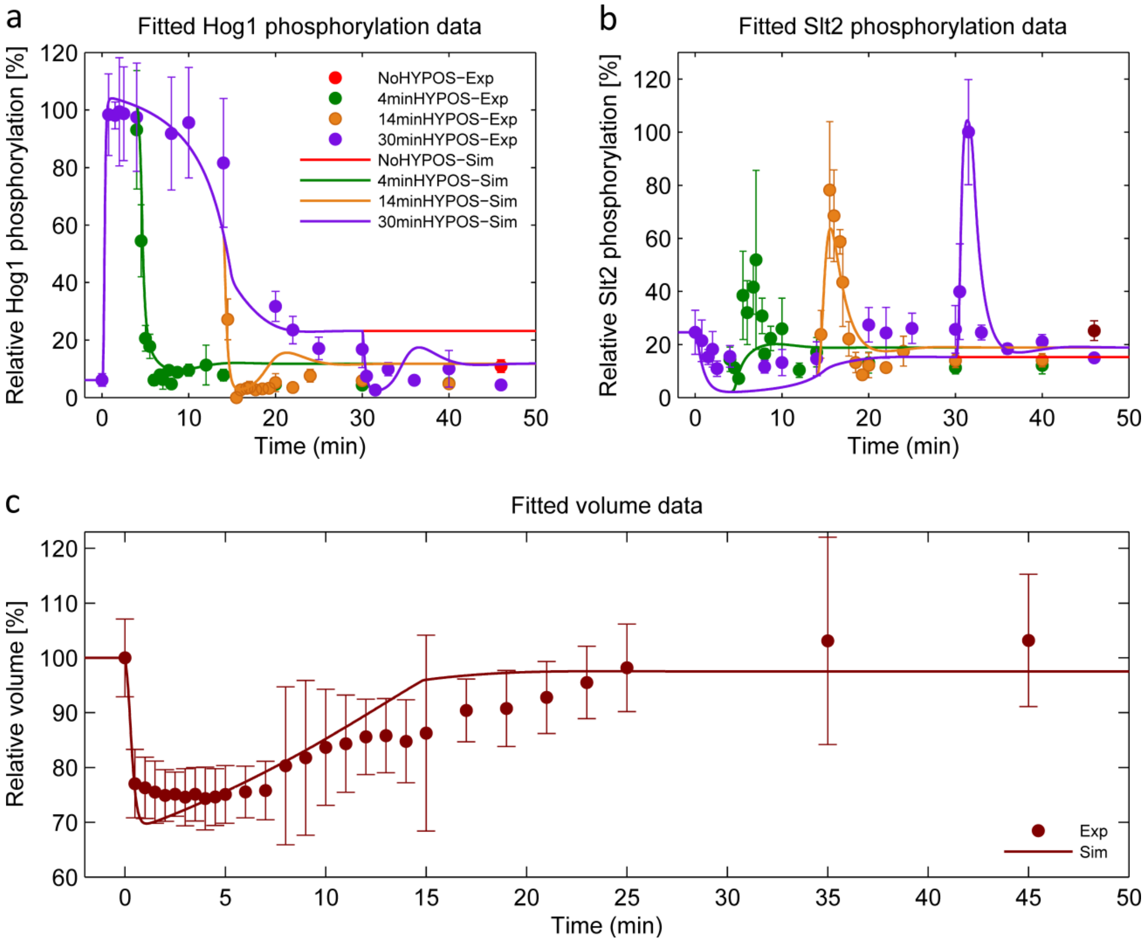

Figure S1: Reproduction of experimental data dedicated for parameter estimation using model with fixed Slt2 activation threshold. Relative Hog1 and Slt2 phosphorylation data and relative single cell volume measurements, used for models parameters estimation, are plotted versus time. Simulations were done using the best ranked model from the ensemble of models with fixed Slt2 activation threshold. Solid lines show model simulations and filled circles (•) show the experimental data (Mean ± SD (n=3)). **a**) Comparison between Hog1 phosphorylation data and respective simulation for 0.8 M sorbitol shock only (NoHYPOS-Ex) and 4’, 14’ and 30’ hypo-shock experiments using the best ranked model (4minHYPOS, 14minHYPOS, 30minHYPOS, respectively). **b**) Comparison between Slt2 phosphorylation data and its simulation for 0.8 M sorbitol shock only, 4’, 14’, 30’ hypo-shock using best ranked model. The selected model can reproduce the 4’ Slt2 activation. **c**) Comparison between relative value of single cell volume measurements and its simulation. The same color code was used for panels A&B.

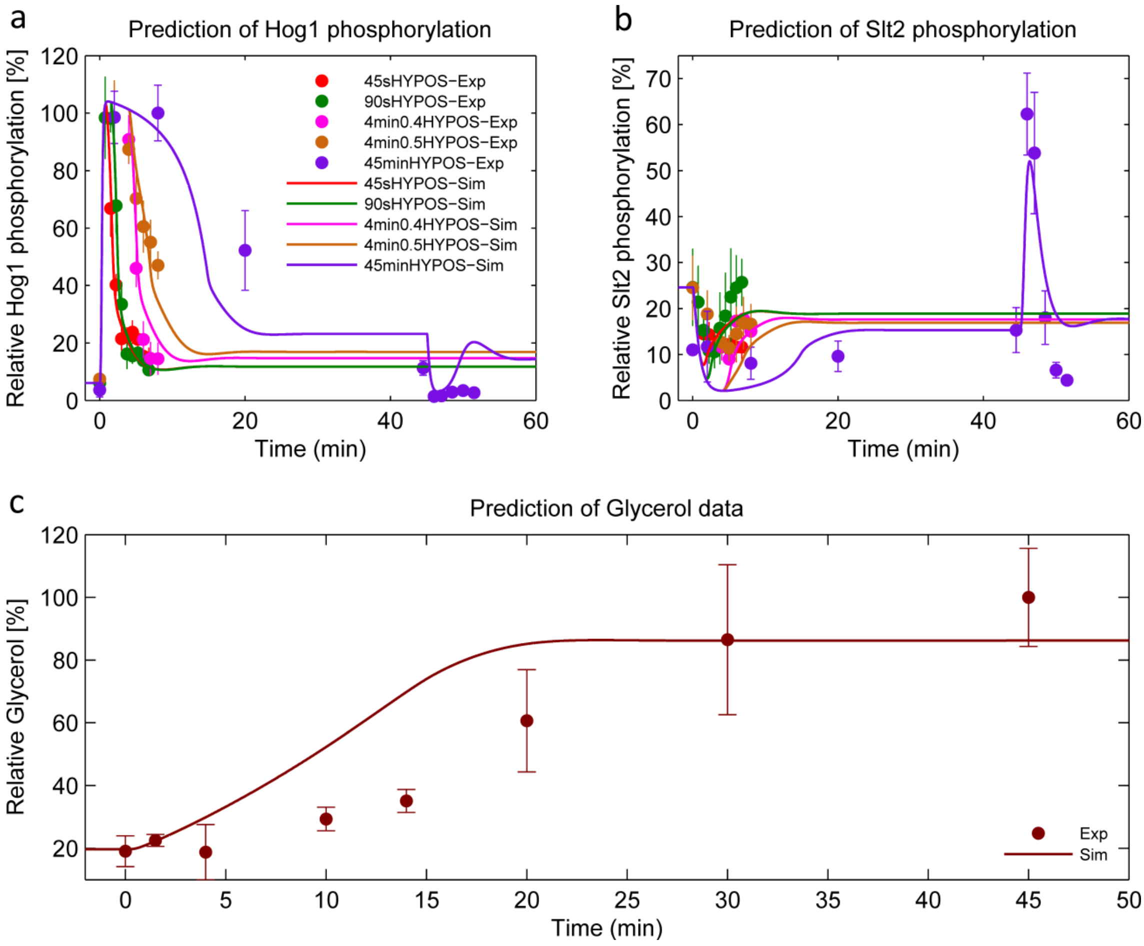

Figure S2: Reproduction of experimental data used for prediction using model with fixed Slt2 activation threshold. Relative Hog1 and Slt2 phosphorylation data and relative value of cellular glycerol measurements, used for prediction, are plotted versus time. Simulations were done using the best ranked model from the ensemble of models with fixed Slt2 activation threshold. Solid lines show model simulations and filled circles (•) show the experimental data (Mean ± SD (n=3)). **a**) Comparison between Hog1 phosphorylation data and its simulation for 0.8 M sorbitol shock with subsequent dilution to 0.27 M sorbitol at 45", 90" and 45' (45SecHYPO-Ex, 90SecHYPO-Ex, 45minHYPO-Ex) and 0.8 M sorbitol shock with subsequent dilution to 0.5 and 0.4 M sorbitol at 4' (4min0.5HYPO-Ex, 4min0.4HYPO-Ex) using the best ranked model. **b**) Comparison between Slt2 phosphorylation data and its simulation for 0.8 M sorbitol shock with subsequent dilution to 0.27 M sorbitol at 45", 90" and 45' and 0.8 M sorbitol shock with subsequent dilution to 0.5 and 0.4 M sorbitol at 4' using best ranked model. **c**) Comparison between relative value of intracellular glycerol content for 0.8 M sorbitol shock and its simulation. We used same color code for panels A&B.

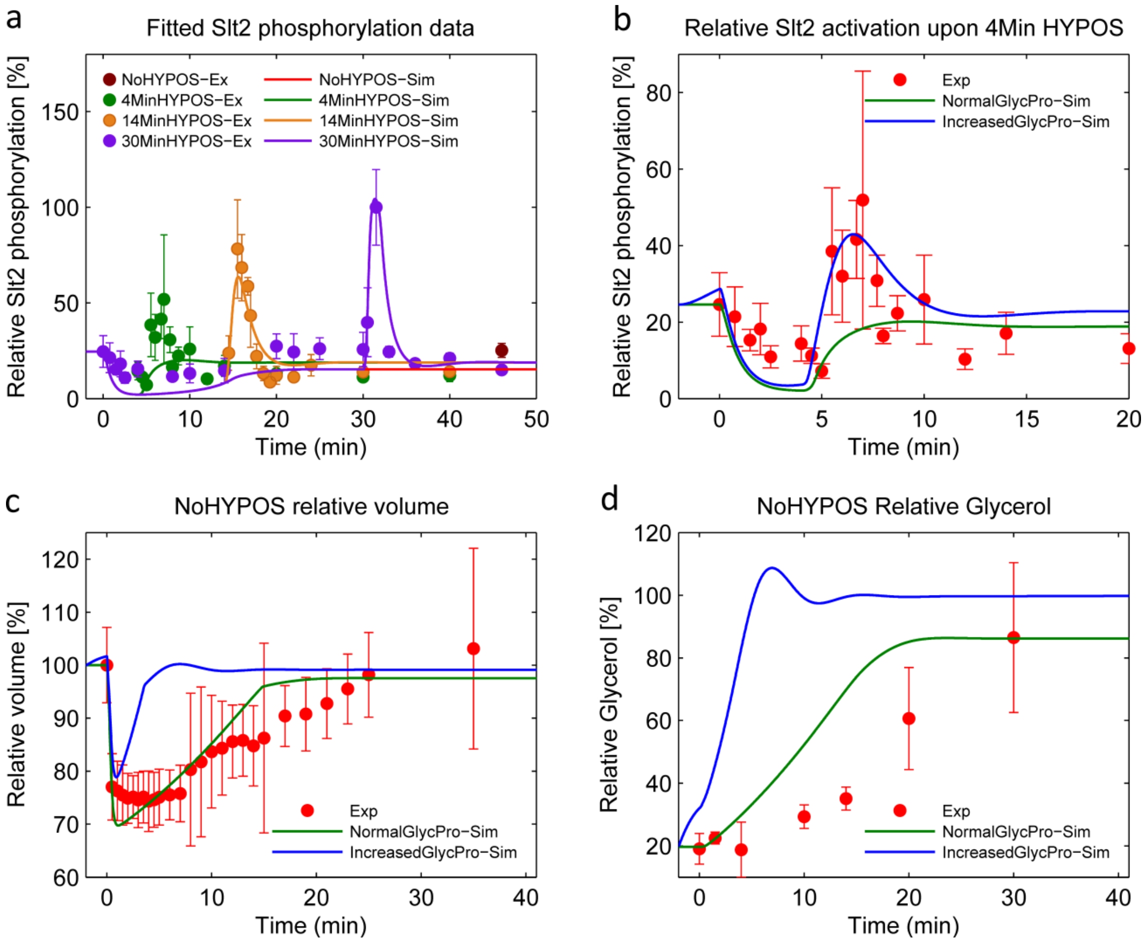

Figure S3: Models were not able to reproduce 4 minute Slt2 phosphorylation peak. Solid lines show model simulations and (•) marks show the experimental data (Mean ± SD (n=3)). **a**) Relative Slt2 phosphorylation data and simulations for 0.8 M sorbitol hyper-osmotic shock with subsequent decrease in external osmolarity to 0.27 M at 4’, 14’, 30’ using best ranked model. The selected model cannot reproduce 4’ Slt2 activation peak. **b**-**d**) Simulation of the selected model with normal (green line) and high (blue line) glycerol production is compared to experimental data. **b**) The model with higher glycerol production rate can reproduce 4’ Slt2 activation, whereas model with normal glycerol production rate cannot. **c**) The model with normal glycerol production rate simulates the relative volume within the measurements error bar, whereas the model with high glycerol production rate fails. **d**) The model with normal glycerol production rate predicts the relative glycerol within the measurements error bar, whereas the model with high glycerol production rate fails.

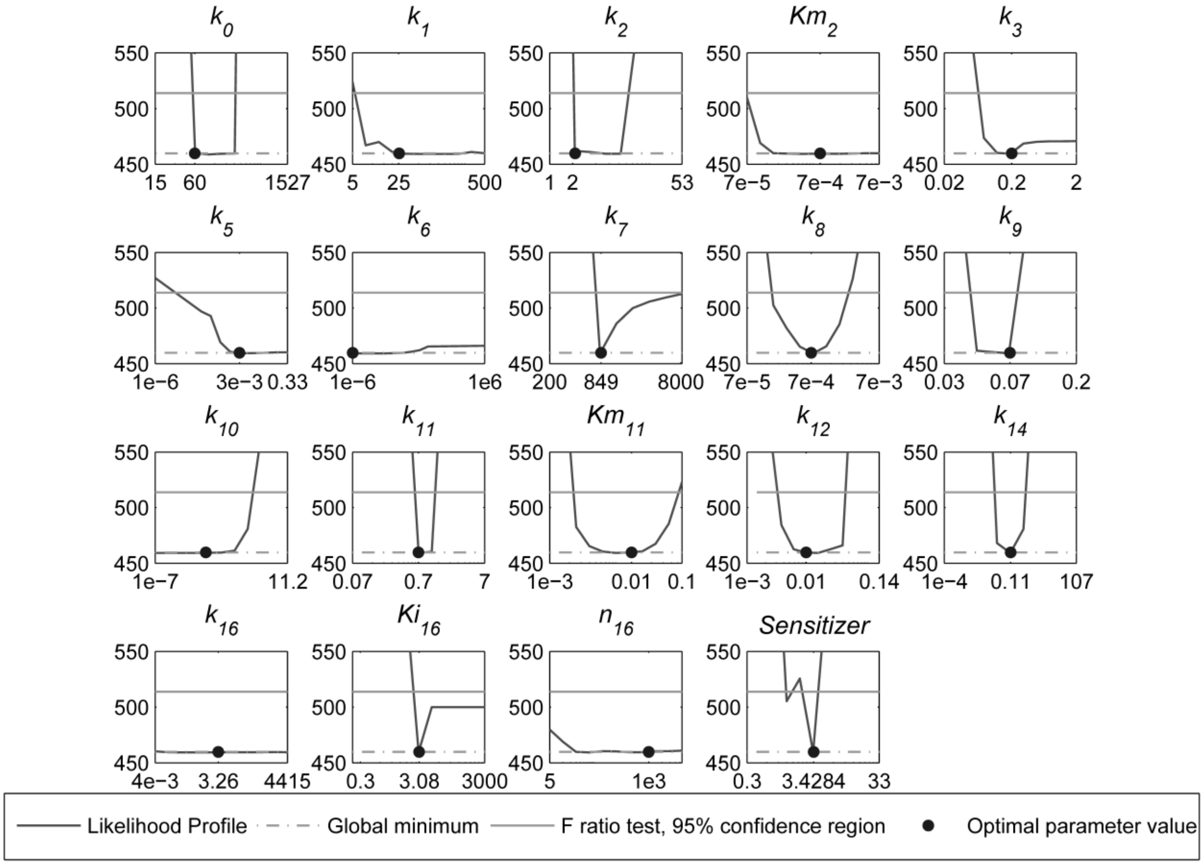

Figure S4: Likelihood profile-based parameter identifiability analysis for the selected model. The SSR after parameter estimation is plotted versus the scanned parameter values (black solid line). 95% confidence region is calculated by F-ratio test (grey solid line). The minimum objective value reached is shown at bottom (grey dashed line) and the corresponding estimated parameter value is shown by a bold dot (•).

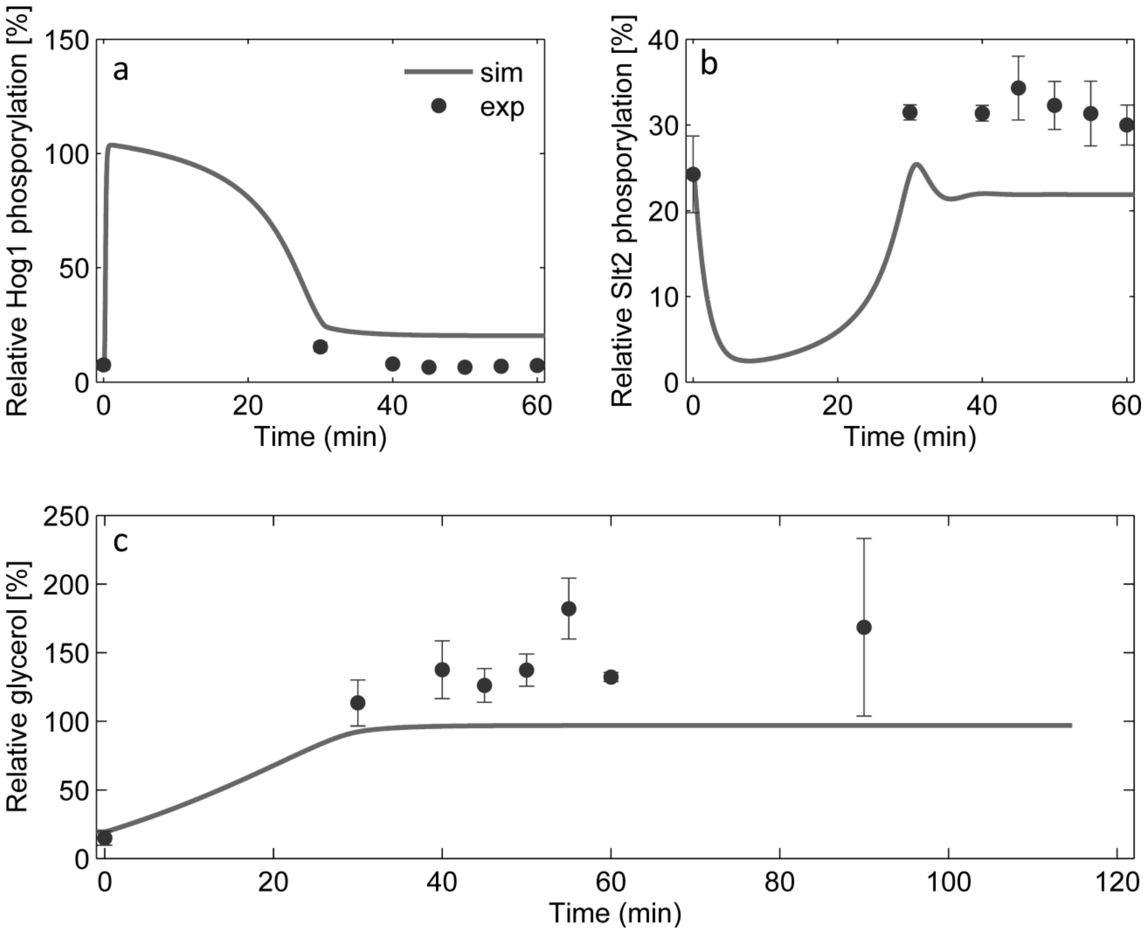

Figure S5: Hog1 and Slt2 Phosphorylation dynamics upon 1.0 M Sorbitol shock.

Relative Slt2 and Hog1 phosphorylation data upon 1.0 M of sorbitol shock are plotted versus time. Solid lines show model simulations and filled circles (•) show the experimental data (Mean ± SD (n=3)). **a** & **b**) Comparison of Slt2 and Hog1 phosphorylation data with their simulation upon 0.8 M of sorbitol shock. Although the overall Slt2 phosphorylation level has increased after sorbitol shock, no marked Slt2 activation is observed in 60 minutes comparing to earlier time-points namely 30 and 40 minutes. This is opposed to the observation made by Garcia et al [6] in which a strong Slt2 activation is observed upon 1.0 M of sorbitol shock. **c**) Intracellular glycerol level after 1.0 M of sorbitol shock is plotted versus time. Again no marked glycerol concentration drop is observed.

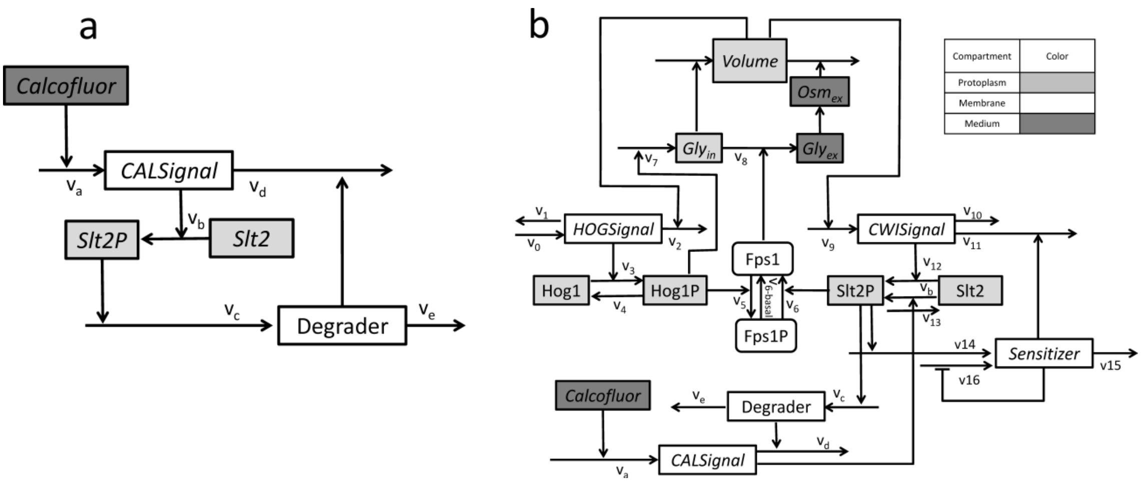

Figure S6: Slt2 activating module.

To activate the Slt2 upon calcofluor exposure we designed a small mathematical module which converts the presence of the calcofluor in the medium to a signal which activates the Slt2. **a**) This mathematical module contains 3 new species, *Calcofluor*, *CALSignal* and *Degrader*. The *CALSignal* is activated in response to calcofluor, v_1, and induces the Slt2 phosphorylation, v_2. Phosphorylated Slt2 activates the species *Degarder*, v_4, which induces the *CALSignal* decay, v4. The *Degrader* is constantly degraded via reaction v_5. **b**) The schematic shows the way that the Slt2 activating module is plugged in to the selected model.

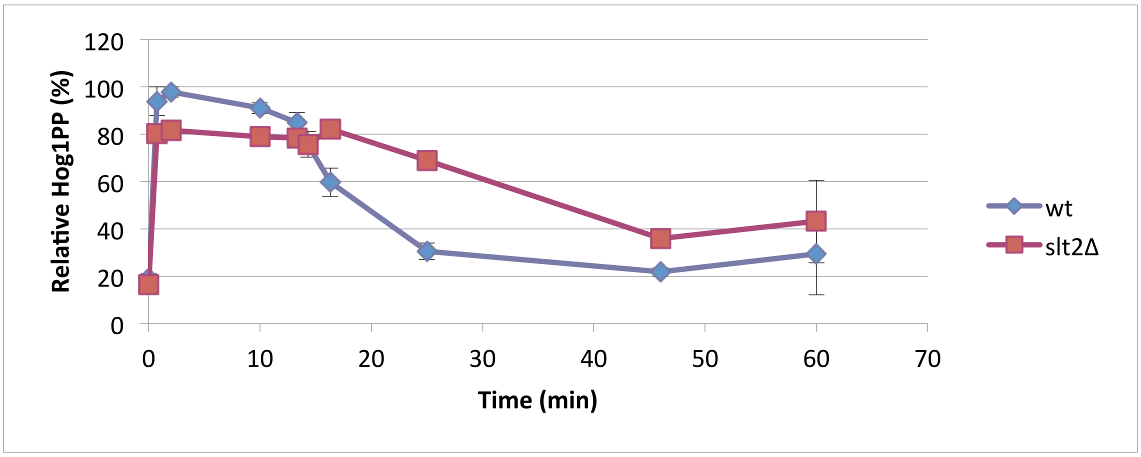

Figure S7: Hog1 response in *slt2Δ* mutants.

Hog1 activation upon 0.8 M of sorbitol stress was monitored in *slt2Δ* mutant cells. Hog1 deactivates slower in *slt2Δ* mutant cells.

Supplementary Tables

Table S1: Models are ranked according to Akaike Information Criterion corrected for small sample size (*AICc*). The data from 45", 90", 45' HYPOS (0.8M to 0.27M sorbitol) and 4' HYPOS (0.8M to 0.4M and 0.5M sorbitol respectively) experiments were not used for parameter estimation (*wSSR*). The best ranked model shows no cross talk between Hog1 and Slt2. Abbreviations: *n*: number of data points, *k*: number of parameters, *wSSR*: weighted sum of squared residuals, *AICc*: Akaike Information Criterion corrected for small smaple size, *AICw*: Akaike weights.

Rank	Model name	HIS	SIH	4MiP	n	k	SSR	*AICc*	*AICc* weight	Cutoff
1st	Model Nr.3				136	14	500.30	594.57	0.963	OK
2nd	Model Nr.4				136	17	499.98	602.20	0.021	NO
3rd	Model Nr.1				136	17	502.78	602.96	0.015	NO
4th	Model Nr.2				136	19	503.00	608.38	0.001	NO

Variable Name			Marker
HIS	SIH	4MiP	
Hog1PP Inhibits Slt2 activation	Slt2P Inhibits Hog1 activation	Model reproduces 4' Slt2 peak	
Hog1PP does not Inhibit Slt2 activation	Slt2P does not inhibit Hog1 activation	Model does not reproduce 4' Slt2 peak	

Table S2: Models are ranked according to Akaike Information Criterion corrected for small sample size (*AICc*). The data from NoHYPOS, 45”, 90”, 45’ HYPOS (0.8M to 0.27M sorbitol) and 4’ HYPOS (0.8M to 0.4M and 0.5M sorbitol respectively) experiments were also used for parameter estimation (wSSR). The best ranked model shows no cross talk between Hog1 and Slt2 again. Abbreviations: *n*: number of data points, *k*: number of parameters, *wSSR*: weighted sum of squared residuals, *AICc*: Akaike Information Criterion corrected for small smaple size, *AICw*: Akaike weights.

Rank	Model name	HIS	SIH	4MiP	n	k	SSR	*AICc*	*AICc* weight	Cutoff
1st	Model Nr.3				234	14	736.98	962.44	0.953	OK
2nd	Model Nr.4				234	17	738.33	969.78	0.024	NO
3rd	Model Nr.1				234	17	739.14	970.04	0.022	NO
4th	Model Nr.2				234	19	744.01	976.29	0.001	NO

Variable Name			Marker
HIS	SIH	4MiP	
Hog1PP Inhibits Slt2 activation	Slt2P Inhibits Hog1 activation	Model reproduces 4’ Slt2 peak	
Hog1PP does not Inhibit Slt2 activation	Slt2P does not inhibit Hog1 activation	Model does not reproduce 4’ Slt2 peak	

Table S3: Models are ranked according to Akaike Information Criterion corrected for small sample size (*AICc*). The data from NoHYPOS, 45”, 90”, 4’ (0.8M sorbitol to 0.4M and 0.5M hypo-osmotic shock respectively) and 45’ hyper-osmotic shock experiments were used for parameter estimation (wSSR). All models with sensitizer component were ranked in top 4 and were able to fit 4’ Slt2 activation peak (4MiP). The best ranked model shows no cross talk between Hog1 and Slt2 (HIS and SIH). Abbreviations: *n*: number of data points, *k*: number of parameters, *wSSR*: weighted sum of squared residuals, *AICc*: Akaike Information Criterion corrected for small sample size, *AICw*: Akaike weights.

Rank	Model name	HIS	SIH	4MiP	*n*	*k*	*wSSR*	*AICc*	*AICc* weight	Cutoff
1st	Model Nr.7				234	19	391.43	826.00	0.594	OK
2nd	Model Nr.8				234	21	385.49	827.23	0.321	OK
3rd	Model Nr.5				234	21	391.31	830.74	0.056	NO
4th	Model Nr.6				234	23	385.29	832.01	0.029	NO
5th	Model Nr.3				234	14	736.98	962.44	0	NO
6th	Model Nr.4				234	17	738.33	969.78	0	NO
7th	Model Nr.1				234	17	739.14	970.04	0	NO
8th	Model Nr.2				234	19	744.01	976.29	0	NO

Variable Name			Marker
HIS	SIH	4MiP	
Hog1PP Inhibits Slt2 activation	Slt2P Inhibits Hog1 activation	Model reproduces 4’ Slt2 peak	
Hog1PP does not Inhibit Slt2 activation	Slt2P does not inhibit Hog1 activation	Model does not reproduce 4’ Slt2 peak	

Table S4: Ordinary differential equation system of the master model.

The equation with the dagger sign (†) is only present in the models with sensitized negative feedback.

ODEs
$\frac{dV_{os}}{dt} = -Lp \cdot Area \cdot \left(Turgor + f_{c2p} \cdot R \cdot T \cdot (Osmo_{ex} - Osmo_{in})\right)$
$\frac{d([Hog1Signal] \cdot V_{membrane})}{dt} = V_{membrane} \cdot (v_0 - v_1 - v_2)$
$\frac{d([Hog1] \cdot V_{os})}{dt} = +V_{os} \cdot (-\{v_{3-a}, v_{3-b}\} + v_4)$
$\frac{d([Hog1PP] \cdot V_{os})}{dt} = +V_{os} \cdot (\{v_{3-a}, v_{3-b}\} - v_4)$
$\frac{d([Fps1])}{dt} = V_{membrane} \cdot (-v_5 + v_6 + v_{6b})$
$\frac{d([Fps1P])}{dt} = V_{membrane} \cdot (v_5 - v_6 - v_{6b})$
$\frac{d([Gly_{in}] \cdot V_{os})}{dt} = +V_{os} \cdot v_7 - v_8$
$\frac{d([Gly_{ex}] \cdot V_{medium})}{dt} = v_8$
$\frac{d([Slt2Signal] \cdot V_{membrane})}{dt} = V_{membrane} \cdot (v_9 - v_{10} - \{v_{11-a}, v_{11-b}\})$
$\frac{d([Slt2] \cdot V_{os})}{dt} = +V_{os} \cdot (-\{v_{12-a}, v_{12-b}\} + v_{13})$
$\frac{d([Slt2PP] \cdot V_{os})}{dt} = +V_{os} \cdot (\{v_{12-a}, v_{12-b}\} - v_{13})$
†$\frac{d([Sensitizer] \cdot V_{membrane})}{dt} = V_{membrane} \cdot (v_{14} + v_{16} - v_{15})$

Table S5: Rate equations of the master model including different model alternatives.

Concentrations are denoted by [] and initial concentration by $[]_0$. The auxiliary variables and parameters are described in Table S7. Bold parameters are free parameters that are estimated from data and their value is reported in Table S8. Reactions with dagger (†) sign are only present in the models with sensitized negative feedback.

Rate	Rate law	Description
V_0	$\boldsymbol{k_0}$	*Hog1Signal* production
V_1	$\boldsymbol{k_1} \cdot [Hog1Sensor]$	*Hog1Signal* degradation
V_2	$\frac{\boldsymbol{v_{max2}} \cdot V_{os} \cdot [Hog1Sensor]}{\boldsymbol{k_{m2}} + [Hog1Sensro]}$	Osmolytically active volume mediated *Hog1Signal* degradation
$V_{3\text{-}a}$	$\boldsymbol{v_{max3}} \cdot [Hog1Sensor] \cdot [Hog1]$	*Hog1* phosphorylation
$V_{3\text{-}b}$	$\frac{\boldsymbol{v_{max3}} \cdot [Hog1Sensor] \cdot [Hog1]}{1 + \boldsymbol{k_{i3}} \cdot [Slt2PP]^{\boldsymbol{n3}}}$	*Slt2PP* mediated *Hog1* activation inhibition
V_4	$k_4 \cdot [Hog1PP]$	*Hog1PP* dephosphorylation
V_5	$k_5 \cdot [Hog1PP] \cdot [Fps1]$	*Hog1PP* mediated *Fps1* closure
V_6	$\boldsymbol{k_6} \cdot [Slt2PP] \cdot [Fps1P]$	*Slt2PP* mediated *Fps1P* dephosphorylation
V_{6b}	$\boldsymbol{k_{6b}} \cdot [Fps1P]$	*Fps1P* dephosphorylation
V_7	$\frac{\boldsymbol{v_{max7}} \cdot [Hog1PP]}{\boldsymbol{k_{m7}} + [Hog1PP]}$	*Hog1PP* mediated Glycerol production
V_8	$Fps1Open \cdot \boldsymbol{k_8} \cdot A \cdot ([Gly_{in}] - [Gly_{ex}])$	*Fps1* facilitated glycerol diffusion
V_9	$\boldsymbol{k_9} \cdot V_{os}$	Osmolytically active volume mediated *Slt2Signal* production
V_{10}	$\boldsymbol{k_{10}} \cdot [Slt2Signal]$	*Slt2Signal* degradation
$V_{11\text{-}a}$	$\frac{\boldsymbol{v_{max11}} \cdot [Slt2Signal]}{\boldsymbol{k_{m11}} + [Slt2Signal]}$	*Slt2Signal* degradation (In models without

		sensitized feedback)
† $V_{11\text{-}b}$	$\frac{\boldsymbol{v_{max11}} \cdot [Sensitizer] \cdot [Slt2Signal]}{\boldsymbol{k_{m11}} + [Slt2Signal]}$	*Sensitizer* mediated *Slt2Signal* degradation
$V_{12\text{-}a}$	$\boldsymbol{k_{12}} \cdot [Slt2Signal] \cdot [Slt2]$	*Slt2* Phosphorylation
$V_{12\text{-}b}$	$\frac{\boldsymbol{v_{max12}} \cdot [Slt2Signal] \cdot [Slt2]}{1 + \boldsymbol{k_{i12}} \cdot [Hog1PP]^{\boldsymbol{n12}}}$	*Hog1PP* mediated *Slt2* activation inhibition
V_{13}	$k_{13} \cdot [Slt2PP]$	*Slt2PP* dephosphorylation
† V_{14}	$\boldsymbol{k_{14}} \cdot [Slt2PP]$	*Slt2PP* mediated *Sensitizer* production
† V_{15}	$k_{15} \cdot [Sensitizer]$	*Sensitizer* degradation
† V_{16}	$\frac{\boldsymbol{k_{16}}}{1 + \left(\frac{Sensitizer}{\boldsymbol{k_{i16}}}\right)^{\boldsymbol{n16}}}$	Auto inhibitory regulated *Sensitizer* production

Table S6: State variables and their initial conditions.

Model's state variables and their initial concentrations are listed below. $[]_0$ indicates initial concentrations. Volumes are in femtolitre (fL), concentrations are in (μmoL/fL). Bold parameters are free parameters that are estimated from data and their value is reported in Table S6. State variables with dagger (†) sign are only present in the models with sensitized negative feedback.

State variable (Compartme-nt)	Initial Concentration	Remark
V_{os}	$V_0 \cdot osf_0$	Osmolytically active volume, derived from a total cell volume of 50 fL and a solid base volume of 41% [7].
Fps1	$\frac{907 \cdot f_{N2\mu M}}{2}$	Aquaglyeroporin *Fps1* is located in cell membrane. This is the open form of Fps1 protein. The total amount of *Fps1* is supposed constant.
Fps1P	$\frac{907 \cdot f_{N2\mu M}}{2}$	Activated *Hog1* phosphorylates *Rgc2* protein which leads in Fps1 closure [8].
Hog1	$6788 \cdot f_{N2\mu M} \cdot (1 - [Hog1PP]_0) \cdot maxHog1nucf_0$	Hog1 is the Map kinase of High Osmolarity Glycerol pathway.
Hog1PP	$6788 \cdot f_{N2\mu M} \cdot [Hog1PP]_0 \cdot maxHog1nucf_0$	Double phosphorylated, i.e. active, *Hog1* MAP kinase. It was derived from data that 6.1 % of the maximal phosphorylation is the steady state

		activation. f_n is the fraction in the nucleus at maximal phohsporylation.
$Slt2$	$3230 \cdot f_{N2\mu M} \cdot (1 - [Slt2PP]_0) \cdot maxHog1nucf_0$	*Slt2* is the MAP Kinase of cell Wall Integrity pathway.
$Slt2PP$	$3230 \cdot f_{N2\mu M} \cdot [Slt2PP]_0 \cdot maxHog1nucf_0$	Double phosphorylated, i.e. active, *Slt2* MAP kinase. It was derived from data that 24.6 % of the maximal phosphorylation is the steady state activation. f_n is the fraction in the nucleus at maximal phohsporylation.
$[\boldsymbol{Gly_{in}}]_\mathbf{0}$	180000	Intracellular glycerol, approximated by assuming a measured value of 0.1 mM/OD in 1 ml sample [9] and assuming 18·106 cells per ml sample culture and an average osmotic cell volume of 29.5 fL, i.e.1/18/29.5·108.
Gly_{ex}	$\frac{[Gly_{in}]_0}{1000}$	Extracellular glycerol, assumed to be 1000 times lower than Gly_{in}. As a consequence, the external

		glycerol acts basically as a sink for the internal glycerol.
† $\boldsymbol{Sensitizer}$	3.42838	A hypothetical entity which modulates *Slt2Signal* degradation rate. The initial concentration of sensitizer is estimated by the model.
HogSignal	$\frac{\boldsymbol{k_0} - V_{os_0} \cdot \boldsymbol{k_2} - \boldsymbol{k_1} \cdot \boldsymbol{k_{m2}}}{2 \cdot k_1} + \frac{\sqrt{\left(\boldsymbol{k_0} - V_{os_0} \cdot \boldsymbol{k_2} - \boldsymbol{k_1} \cdot \boldsymbol{k_{m2}}\right)^2 + 4 \cdot \boldsymbol{k_0} \cdot \boldsymbol{k_1} \cdot \boldsymbol{k_{m2}}}}{2 \cdot k_1}$	A hypothetical entity which triggers *Hog1* activation.
† *Slt2Signal*	$\frac{\boldsymbol{k_9} \cdot V_{os_0} - \boldsymbol{k_{11}} \cdot [Sensitizer]_0 - \boldsymbol{k_{10}} \cdot \boldsymbol{k_{m11}}}{2 \cdot k_1} + \frac{\sqrt{\left(\boldsymbol{k_{11}} \cdot [Sensitizer]_0 - \boldsymbol{k_{10}} \cdot \boldsymbol{k_{m11}} - \boldsymbol{k_9} \cdot V_{os_0}\right)^2 + 4 \cdot \boldsymbol{k_{10}} \cdot V_{os_0} \cdot \boldsymbol{k_9} \cdot \boldsymbol{k_{m11}}}}{2 \cdot k_1}$	A hypothetical entity which triggers *Slt2* activation.
Slt2Signal	$\frac{\boldsymbol{k_9} \cdot V_{os_0} - \boldsymbol{k_{11}} - \boldsymbol{k_{10}} \cdot \boldsymbol{k_{m11}}}{2 \cdot k_1} + \frac{\sqrt{\left(\boldsymbol{k_{11}} - \boldsymbol{k_{10}} \cdot \boldsymbol{k_{m11}} - \boldsymbol{k_9} \cdot V_{os_0}\right)^2 + 4 \cdot \boldsymbol{k_{10}} \cdot V_{os_0} \cdot \boldsymbol{k_9} \cdot \boldsymbol{k_{m11}}}}{2 \cdot k_1}$	A hypothetical entity which triggers *Slt2* activation.

Table S7: Auxiliary variables, physical quantities and their Definition/value.

Concentrations are denoted by [] and $[]_0$ denotes the initial concentration. Volumes are in femtolitre (fL), concentrations are in (μmol/fL).

Variable/Parameter	Definition/Value	Remark
$\boldsymbol{V_b}$	$V_0 \cdot f_{min}$	Solid or minimal volume of the cell.
$\boldsymbol{V}$	$V_{os} + V_b$	Total cell volume.
$\boldsymbol{V_{os_0}}$	$V_0(1 - f_{min})$	Initial osmotically active volume.
$\boldsymbol{V_{P=0}}$	$V_0 e^{\frac{P_0}{\varepsilon}}$	Non-turgid volume.
$\boldsymbol{A}$	$(36\pi)^{1/3} V^{2/3}$	Total cell surface area.
$\boldsymbol{f_{N2\mu M}}$	$10^{21} mol^{-1} V_{os_0}^{-1}$	Factor converting number of molecules in μM concentrations per cell.
$\boldsymbol{c_0^i}$	$c_0^e + \frac{P_0}{f_{c2p} RT}$	Initial total cellular osmolyte concentration.
$\boldsymbol{c_0^{in}}$	$c_0^i - [Gly_{in}]$	Initial non-permeable cellular osmolyte concentration.
$\boldsymbol{c_n^e}$	$\begin{cases} 0 & t < t_{off} \\ s1 \cdot \left(1 - e^{\frac{t_{off} - t}{t_m}}\right) & t_{off} < t < t_{off} + t_s \\ (s1 - s2) \cdot e^{\frac{t_{off} + t_s - t}{t_m}} & else \end{cases}$	Osmotic sorbitol shock. Starts at time t_s and has a certain mixing time t_m.
$\boldsymbol{c_0^e}$ [μM]	260000	Initial osmolarity of the medium [7].
$\boldsymbol{t_{off}}$ [s]	120	Time [s] before first osmotic stress.
$\boldsymbol{t_s}$ [s]	840	Time [s] between two consecutive osmotic stresses.
$\boldsymbol{t_m}$ [s]	10	Mixing time [s] of sorbitol in the medium.
S1[μM]	800000	Sorbitol concentration, in cell culture medium,

		for first osmotic stress.
S2[μM]	270000	Sorbitol concentration, in cell culture medium, for second osmotic stress.
$\boldsymbol{Osm_{in}}$	$[Gly_{in}] + \frac{c_0^{in} V_{os_0}}{V_{os}}$	Intracellular osmolytically active concentration.
$\boldsymbol{Osm_{ex}}$	$c_0^e + c_n^e + [Gly_{ex}] - [Gly_{ex}]_0$	Extracellular osmolytically active concentration.
$\boldsymbol{Turgor}$	$\begin{cases} \varepsilon \cdot ln\left(\frac{V}{V_P}\right) & for\ \ V > V_{P=0} \\ 0 & else \end{cases}$	Turgor pressure [MPa].
R [J/mol/K]	8.314	Gas constant.
T [K]	303.15	Temperature in kelvin corresponds to 30°C.
Mol	$6.022 \cdot 10^{23}$	Mole number.
$\boldsymbol{f_{c2p}}$	10^{-9}	Factor converting concentrations in M to pressures in MPa.
$\boldsymbol{L_p}$ [μm/Mpa/s]	0.013	Hydraulic conductivity (estimate from data from [10]).
$\boldsymbol{P_0}$ [MPa]	0.61	Initial turgor pressure [7].
ε	14.3	Membrane rigidity [7].
$f_{\min}$	0.41	Minimal cell volume (as fraction of total) [7].
$\boldsymbol{f_n}$	0.8	Fraction of activated Hog1 molecule in the nucleus upon maximal activation.
$\boldsymbol{V_0}$ [fL]	50	Initial total cell volume.
$\boldsymbol{V_{medium}}$ [fL]	$1000 * V_0$	External volume.
$\boldsymbol{Hog1_t}$ [μM]	0.3821	$6788 \cdot f_{n2\mu M}$: molecule numbers from http://yeastgfp.yeastgen

		ome.org/
$\boldsymbol{Fps1_t}$ [μM]	0.051	$907 f_{n2\mu M}$: molecule numbers from http://yeastgfp.yeastgenome.org/

Table S8: Reaction rate constants and model parameters.

$[]_0$ indicates initial concentration (µmol/L). The volume is in femtolitre (fL), and the concentration is µmol/L, mass is in grams and time in seconds. Variables with dagger sign (†) are only present in the models with sensitized negative feedback.

Parameter	Value	Description	Method
k_0	59.901	*Hog1Signal* production rate constant.	Estimated
k_1	25.4711	*Hog1Signal* degradation rate constant.	Estimated
v_{max2}	2.15338	Volume mediated *Hog1Signal* degradation v_{max}.	Estimated
k_{m2}	0.000939165	Volume mediated *Hog1Signal* degradation Michaelis constant.	Estimated
k_3	0.231769	*Hog1* phosphorylation rate constant.	Estimated
k_4	$\frac{k_3 \cdot [Hog1Signal]_0 \cdot [Hog1]_0}{[Hog1PP]_0 \cdot \left[1 + K_{i3} \cdot [Slt2PP]_0{}^{n3}\right]}$	*Hog1PP* dephosphorylation rate constant, calculated using steady state assumption.	Calculated
k_5	0.12348	*Hog1PP* mediated *Fps1* closure rate constant.	Estimated
k_6	1.1077e-06	*Slt2PP* mediated *Fps1* depohsphorylation (opening).	Calculated
k_{6b}	$\frac{k_5 \cdot [Hog1PP]_0 \cdot [Fps1]_0 - k_6 \cdot [Slt2PP]_0 \cdot [Fps1P]_0}{[Fps1P]_0}$	*Slt2PP* independent (Basal) *Fps1* dephosphorylation.	Estimated
v_{max7}	849.986	*Hog1PP* mediated glycerol production v_{max}.	Estimated

k_{m7}	$\frac{v_{max7} \cdot [Hog1PP]_0 \cdot V_{os_0}}{[Fps1_{open}]_0 \cdot k_8 \cdot A \cdot ([Gly_{in}]_0 - [Gly_{ex}]_0 - [Hog1PP]_0}$	*Hog1PP* mediated glycerol production Michaelis constant, calculated using steady state assumption.	Calculated
k_8	0.000776772	*Fps1* facilitated glycerol diffusion constant.	Estimated
k_9	0.0772144	Volume mediated *Slt2Signal* production rate constant.	Estimated
k_{10}	0.000131323	*Slt2Signal* degradation rate constant.	Estimated
v_{max11}	0.713377	*Sensitizer/Slt2PP* mediated *Slt2Signal* degradation v_{max}.	Estimated
k_{m11}	0.0180575	*Sensitizer/Slt2PP* mediated *Slt2Signal* degradation k_m.	Estimated
k_{12}	0.00929813	*Slt2Signal* mediated *Slt2* phosphorylation rate constant.	Estimated
k_{13}	$\frac{v_{max12} \cdot [Slt2Signal]_0 \cdot [Slt2]_0}{[Slt2PP]_0 \cdot \left(1 + K_{i12} \cdot {[Hog1PP]_0}^{n12}\right)}$	*Slt2PP* dephosphorylation rate constant, calculated using steady state assumption.	Calculated
† k_{14}	0.113591	*Slt2PP* mediated *sensitizer* production rate constant.	Estimated
† k_{15}	$\frac{k_{14} \cdot [Slt2PP]_0 + \frac{v_{max16}}{1+\left(\frac{[Sensitizer]_0}{K_{i16}}\right)^{n16}}}{[Sensitizer]_0}$	*Sensitizer* degradation rate constant, calculated using steady state assumption.	Calculated

† v_{max16}	1	*Sensitizer* Auto inhibitory feedback v_{max}.	set
† K_{i16}	3.08897	*Sensitizer* Auto inhibitory feedback constant.	Estimated
† n_{16}	999.898	*Sensitizer* Auto inhibitory feedback power.	Estimated

Supplementary References:

1 Hoops, S. *et al.* COPASI--a COmplex PAthway SImulator. *Bioinformatics* **22**, 3067-3074, doi:10.1093/bioinformatics/btl485 (2006).
2 Schaber, J. *et al.* Automated ensemble modeling with modelMaGe: analyzing feedback mechanisms in the Sho1 branch of the HOG pathway. *PLoS One* **6**, e14791, doi:10.1371/journal.pone.0014791 (2011).
3 Burnham, K. P. & Anderson, D. R. *Model Selection and Multi-Model Inference: A Practical Information-Theoretic Approach*. (Springer, 2010).
4 Raue, A. *et al.* Structural and practical identifiability analysis of partially observed dynamical models by exploiting the profile likelihood. *Bioinformatics* **25**, 1923-1929, doi:10.1093/bioinformatics/btp358 (2009).
5 Schaber, J. Easy parameter identifiability analysis with COPASI. *Biosystems*, doi:10.1016/j.biosystems.2012.09.003 (2012).
6 Garcia-Rodriguez, L. J., Valle, R., Duran, A. & Roncero, C. Cell integrity signaling activation in response to hyperosmotic shock in yeast. *FEBS Lett* **579**, 6186-6190, doi:10.1016/j.febslet.2005.10.001 (2005).
7 Schaber, J. *et al.* Biophysical properties of Saccharomyces cerevisiae and their relationship with HOG pathway activation. *Eur Biophys J* **39**, 1547-1556, doi:10.1007/s00249-010-0612-0 (2010).
8 Lee, J. *et al.* MAPK Hog1 closes the S. cerevisiae glycerol channel Fps1 by phosphorylating and displacing its positive regulators. *Genes Dev* **27**, 2590-2601, doi:10.1101/gad.229310.113 (2013).
9 Klipp, E., Nordlander, B., Kruger, R., Gennemark, P. & Hohmann, S. Integrative model of the response of yeast to osmotic shock. *Nat Biotechnol* **23**, 975-982, doi:10.1038/nbt1114 (2005).
10 Eriksson, E. *et al.* A microfluidic device for reversible environmental changes around single cells using optical tweezers for cell selection and positioning. *Lab Chip* **10**, 617-625, doi:10.1039/b913587a (2010).
